# Clinical significance of chronic bronchitis in different racial groups

**DOI:** 10.1186/s12890-024-03100-y

**Published:** 2024-06-17

**Authors:** Joon Young Choi, Kwang Ha Yoo, Ki-Suck Jung, Victor Kim, Chin Kook Rhee

**Affiliations:** 1 Division of Pulmonary and Critical Care Medicine, Department of Internal Medicine, Incheon St. Mary’s Hospital, College of Medicine, The Catholic University of Korea, Seoul, Republic of Korea; 2 Division of Pulmonary, Allergy and Critical Care Medicine, Department of Internal Medicine, Konkuk University School of Medicine, Seoul, Republic of Korea; 3 Division of Pulmonary, Allergy and Critical Care Medicine, Hallym University Sacred Heart Hospital, Hallym University Medical School, Anyang, Republic of Korea; 4 Department of Thoracic Medicine and Surgery, Lewis Katz School of Medicine at Temple University, 3401 North Broad Street, 785 Parkinson Pavilion, Philadelphia, PA 19140 USA; 5 Division of Pulmonary and Critical Care Medicine, Department of Internal Medicine, Seoul St. Mary’s Hospital, College of Medicine, The Catholic University of Korea, 222 Banpo-daero, Seocho-gu, Seoul, 06591 Republic of Korea

**Keywords:** Chronic obstructive pulmonary disease, Chronic bronchitis, COPDGene, KOCOSS, Racial difference

## Abstract

**Backgrounds:**

Limited data are available on racial differences in the clinical features of chronic bronchitis (CB) patients with chronic obstructive pulmonary disease (COPD). In this study, we aimed to compare clinical features among CB patients of different races. We also analyzed the clinical significance of CB, defined classically and based on the COPD Assessment Test (CAT), to validate the CAT-based definition.

**Methods:**

We analyzed patient data extracted from the Korean COPD Subgroup Study (KOCOSS) cohort (2012–2021) and US Genetic Epidemiology of COPD (COPDGene) study (2008–2011). We compared clinical characteristics among CB and non-CB patients of three different races using two CB definitions.

**Results:**

In this study, 3,462 patients were non-Hispanic white (NHW), 1,018 were African American (AA), and 1,793 were Asian. The proportions of NHW, AA, and Asian patients with CB according to the classic definition were 27.4%, 20.9%, and 10.7%, compared with 25.2%, 30.9%, and 23.0% according to the CAT-based definition, respectively. The risk of CB prevalence was highest in NHW and lowest in Asian COPD patients. Among all races, CB patients were more likely to be current smokers, have worse respiratory symptoms and poorer health-related quality of life (HrQoL), and to have decreased lung function and exercise capacity. Most of these characteristics showed similar associations with the outcomes between the two definitions of CB. A binominal regression model revealed that CB patients of all races had an increased risk of future exacerbations according to both CB definitions, except for Asian patients with classically defined CB.

**Conclusions:**

The presence of CB was associated with worse respiratory symptoms, HrQoL, exercise capacity and lung function, and more exacerbations, regardless of race or CB definition. The CAT-based definition may be more useful for assessing the risk of future exacerbations in Asian COPD patients.

**Supplementary Information:**

The online version contains supplementary material available at 10.1186/s12890-024-03100-y.

## Introduction

Chronic obstructive pulmonary disease (COPD) is a heterogeneous disease with various pathological, radiological, and clinical features [1]. Recent studies have identified various clinically important phenotypes of COPD, including chronic bronchitis (CB), emphysema, rapid decliner, frequent exacerbator, and asthma-COPD overlap (ACO) [2–4]. The clinical importance of CB has been studied extensively; it is associated with poor health-related quality of life (HrQoL) [5, 6], decreased lung function [7, 8] and frequent exacerbations [9–11].

CB is characterized by the long-term presence of cough and sputum. It is classically defined as frequent cough and sputum for at least 3 months annually during 2 consecutive years, as proposed by the American Thoracic Society (ATS) in 1978 [12]. However, this definition may be too complex for some patients and could lead to recall bias [13]. Hence, various attempts have been made to overcome these limitations. Kim et al. used St. Georges’ Respiratory Questionnaire (SGRQ) subscales for cough and sputum to define CB, which demonstrated potential as an alternative means of diagnosis [14, 15]. We proposed that CB be diagnosed based on the cough (CAT1) and sputum (CAT2) subscales of the COPD Assessment Test (CAT) [16]. CB patients diagnosed using the CAT score showed comparable clinical characteristics and outcomes to those diagnosed using the classical approach in a South Korean cohort [9, 13, 16]. However, the CAT-based definition must be validated in other racial groups before it can be used worldwide.

In this study, we compared different risk of the CB prevalence and clinical features of CB patients of three different races (non-Hispanic white [NHW], African-American [AA], and Asian). We assessed whether the presence of CB increased the risk of future exacerbations in the three races. Furthermore, we analyzed the clinical significance of CB defined using both the classical and CAT-based definitions, to validate the utility of the latter in different races.

## Methods

### Study population and data collection

We analyzed baseline demographic and clinical data from the Korean COPD Subgroup Study (KOCOSS) cohort (2012–2021) and phase I-II of the US Genetic Epidemiology of COPD (COPDGene) study (2008–2011). The KOCOSS is a prospective, multicenter, observational COPD cohort study involving 54 referral centers in South Korea, and has been ongoing since April 2012 [17]. The inclusion criteria were age ≥ 40 years and post-bronchodilator forced expiratory volume in 1 s/forced vital capacity (FEV_1_/FVC) < 0.7. We analyzed 1-year follow-up data for exacerbations. The COPDGene study is a prospective, multicenter, observational US cohort study including NHW and AA patients aged ≥ 45 years with a smoking history of ≥ 10 pack-years [18, 19]. We also analyzed 1-year exacerbation data from phase II of the COPDGene study (2013–2017). We merged the two cohort databases and excluded patients with a smoking history < 10 pack-years or no airflow limitation.

### Clinical parameters

We collected baseline demographic data including age, sex, race, smoking history, and body mass index (BMI). Comorbidities including diabetes mellitus, hypertension, history of myocardial infarction, heart failure, gastroesophageal reflux, and stroke or transient ischemic attack were obtained. Scores reflecting symptoms and functional exercise capacity, including the modified Medical Research Council (mMRC) dyspnea score, SGRQ score, and CAT score, as well as the 6-min walk distance test (6MWT), were obtained. Depression symptoms was assessed by the Beck Depression Inventory (BDI) in the KOCOSS cohort (BDI score ≥ 10) and the Hospital Anxiety and Depression Scale (HADS)-Depression (HADS-D score ≥ 8) in the COPDGene cohort [20, 21]. Anxiety was assessed by the Beck Anxiety Inventory (BAI) in the KOCOSS cohort (BAI score ≥ 8) and HADS-Anxiety (HADS-A score ≥ 8) in the COPDGene cohort [20, 21]. Presence of emphysema in baseline chest computed tomography (CT) was collected. All demographic data in COPDGene used in our study was based on phase I database, except for CAT score and HADS score, which were based on phase II database. All demographic data in KOCOSS used in this study were collected at the baseline time point. Data on the baseline peripheral eosinophil count, pulmonary function test results, use of medications, and exacerbation history were obtained. Exacerbations were defined as acute respiratory symptoms that required additional medication, such as systemic steroids or antibiotics. Severe exacerbations were defined as exacerbations that required an emergency room encounter or hospitalization.

### Definitions of CB

Two definitions of CB were considered. The classic definition is cough and sputum for > 3 months annually during 2 consecutive years [12]. CB was also defined based on two CAT subscales: CAT1 (cough) score ≥ 3 and CAT2 (sputum) score ≥ 3 [9, 13, 16].

### Statistical analysis

We compared clinical characteristics between non-CB and CB patients in three racial groups (NHW, AA, and Asian) using the two CB definitions. Quantitative variables are presented as mean ± standard deviation and were analyzed with Student’s *t*-test or ANOVA. Categorical variables are presented as frequencies (percentages) and were evaluated with the chi-square test. The risk of CB prevalence by race was analyzed with a multiple logistic regression model; covariates including age, sex, smoking status, baseline post-bronchodilator FEV1 (%) and history of exacerbation were adjusted. The frequency of exacerbation at the 1-year follow-up was analyzed using a negative binomial regression model. The risk of severe exacerbation at the 1-year follow-up was also analyzed with multiple logistic regression model. Covariates including age, sex, smoking status, baseline post-bronchodilator FEV_1_ (%), and the presence of exacerbations in the previous year were adjusted for in the regression models. We used exacerbation frequency at the 1-year follow-up as the outcome variable in the KOCOSS cohort, with study enrollment in the preceding year as the covariate. In the COPDGene study, we used annual exacerbation frequency during phase II as the outcome variable, with that in phase I as the covariate.

All statistical analyses were performed with R software (version 3.6.3; R Foundation for Statistical Computing, Vienna, Austria). *P*-values < 0.05 were considered significant.

## Results

### Baseline characteristics

A total of 1,793 patients in the KOCOSS cohort and 4,480 in the COPDGene cohort were included in this study (Figure S1). Among the patients in the COPDGene study, 3,462 were NHW and 1,018 were AA. The baseline characteristics of the three racial groups are presented in Table 1. The AA patients were younger and more likely to be current smokers with worse symptoms, HRQoL and exercise capacity, as evaluated by the mMRC, SGRQ, and 6MWT. The Asian patients were mostly male, least likely to be current smokers, and had a lower BMI with lower symptom burden and HRQoL according to the mMRC and SGRQ. Emphysema was more likely to be present in NHW compared to AA or Asian group. Lung function was worst in the NHW group, and the rate of past exacerbations was lowest in the Asian group.

**Table 1:** Baseline characteristics of the COPDGene and KOCOSS participants

	NHW (*n* = 3,462)	AA (*n* = 1,018)	Asian (*n* = 1,793)	*P*-value
Age (year)	64.3 ± 8.3	58.6 ± 8.2	69.0 ± 7.7	< 0.01
Sex (male)	1948 (56.3%)	556 (54.6%)	1742 (97.2%)	< 0.01
Smoking status				< 0.01
-Ex-smoker	2185 (63.1%)	352 (34.6%)	1275 (71.1%)	
-Current smoker	1277 (36.9%)	666 (65.4%)	518 (28.9%)	
Smoking history (pack-years)	54.3 ± 27.6	42.2 ± 23.6	44.6 ± 24.3	< 0.01
BMI	27.9 ± 5.9	27.9 ± 6.6	22.9 ± 3.4	< 0.01
Comorbidities				
- DM	394 (11.4%)	155 (15.2%)	310 (17.3%)	< 0.01
- HTN	1617 (46.7%)	544 (53.4%)	712 (39.7%)	< 0.01
- Myocardial infarction	286 (8.3%)	59 (5.8%)	73 (4.1%)	< 0.01
- Heart failure	154 (4.4%)	51 (5.0%)	60 (3.3%)	0.07
- GERD	1107 (32.0%)	198 (19.4%)	219 (12.2%)	< 0.01
- Stroke or TIA	197 (5.7%)	57 (5.6%)	11 (2.5%)	0.02
mMRC	1.9 ± 1.5	2.1 ± 1.5	1.3 ± 0.9	< 0.01
Total SGRQ score	35.9 ± 22.4	40.1 ± 24.2	31.7 ± 18.9	< 0.01
- Symptom	42.1 ± 25.7	44.0 ± 26.3	41.7 ± 19.8	0.82
- Activity	50.6 ± 29.0	55.5 ± 30.0	43.1 ± 23.6	< 0.01
- Impact	25.7 ± 21.2	30.1 ± 24.0	22.1 ± 19.6	< 0.01
CAT score	14.2 ± 8.4	16.5 ± 8.8	14.5 ± 8.0	0.30
- CAT1 (cough)	2.1 ± 1.4	2.3 ± 1.4	1.7 ± 1.4	< 0.01
- CAT2 (sputum)	1.8 ± 1.4	1.9 ± 1.5	2.0 ± 1.4	< 0.01
6MWT (m)	387.4 ± 121.6	336.5 ± 125.2	383.4 ± 114.0	< 0.01
CB (classic definition)	949 (27.4%)	213 (20.9%)	188 (10.7%)	< 0.01
CB (CAT definition)	468 (25.2%)	158 (30.9%)	411 (23.0%$)	< 0.01
Depression	208 (11.5%)	56 (11.0%)	286 (27.5%)	< 0.01
Anxiety	237 (13.1%)	80 (15.7%)	170 (19.4%)	< 0.01
Blood eosinophil count	202.0 ± 157.1	167.2 ± 136.1	230.7 ± 257.7	< 0.01
GOLD stage				< 0.01
- I (FEV1 ≥ 80%)	609 (17.6%)	178 (17.5%)	210 (11.7%)	
- II (FEV1 50–80%)	1444 (41.7%)	482 (47.3%)	981 (54.7%)	
- III (FEV1 30–50%)	918 (26.5%)	244 (24.0%)	505 (28.2%)	
- IV (FEV1 < 30%)	491 (14.2%)	114 (11.2%)	96 (5.4%)	
postBD FEV1 (L)	1.7 ± 0.8	1.6 ± 0.7	1.7 ± 0.6	0.56
postBD FEV1 (%)	56.9 ± 22.9	59.1 ± 22.1	58.3 ± 18.1	0.01
postBD FVC (L)	3.2 ± 1.1	2.8 ± 1.0	3.4 ± 0.8	< 0.01
postBD FVC (%)	81.5 ± 19.9	82.8 ± 21.2	81.7 ± 16.4	0.58
postBD FEV1/FVC	0.51 ± 0.14	0.55 ± 0.12	0.50 ± 0.12	0.08
DLco	67.4 ± 23.0	57.2 ± 20.3	63.0 ± 20.7	< 0.01
Emphysema on CT	1989 (70.1%)	448 (49.1%)	446 (48.3%)	< 0.01
Medications				< 0.01
- no inhaler	404 (30.6%)	132 (34.3%)	420 (23.4%)	
- LABA or LAMA	164 (12.4%)	51 (13.2%)	438 (24.4%)	
- LABA/LAMA	14 (1.1%)	1 (0.3%)	325 (18.1%)	
- ICS/LABA	210 (15.9%)	71 (18.4%)	214 (11.9%)	
- ICS/LABA/LAMA	528 (40.0%)	130 (33.8%)	396 (22.1%)	
Past exacerbation	1218 (35.2%)	319 (31.3%)	353 (20.3%)	< 0.01
Past severe exacerbation	594 (17.2%)	282 (27.7%)	171 (9.9%)	< 0.01.

Data are presented as n (%) or mean ± SD

Demographic data in COPDGene was based on phase I database, except for CAT score and HADS score, which were based on phase II database

All demographic data in KOCOSS was based on the data at the baseline of the study

BMI Body mass index, DM Diabetes mellitus, HTN Hypertension, GERD Gastroesophageal reflux disease, CT Computed tomography, BDI Beck Depression Inventory, BAI Beck Anxiety Inventory, mMRC modified Medical Research Council,, CAT COPD Assessment Test, 6MWT 6-minute walk distance test, ACO Asthma-COPD overlap

LAMA long-acting muscarinic antagonist, LABA long-acting beta2-agonist, ICS inhaled corticosteroids

### Prevalence of CB between races

By classic definition of CB, NHW showed highest of CB prevalence followed by AA compared to Asian patients (Table S1). By CAT definition. NHW showed higher of CB prevalence compared to Asian, but there were no significant differences between the AA and Asian groups.

### Differences in clinical characteristics between the non-CB and CB groups

In total, 21.6% of the patients were diagnosed with CB using the classical definition, compared with 24.9% using the CAT-based definition (Table 2). The CB patients were younger, more likely to be current smokers, had more smoking pack-years, and had more severe symptoms according to the mMRC, SGRQ, CAT, and 6MWT. FEV_1_ was lower in the CB patients regardless of CB definition, and they had more frequent exacerbations and more severe exacerbation events. Additionally, the use of triple therapy was more prevalent among CB patients.

**Table 2:** Differences of clinical characteristics between CB and non-CB according to 2 different definitions

	Classic definition			CAT definition		
	Non-CB (*n* = 4890, 78.4%)	CB (*n* = 1350, 21.6%)	*P*-value	Non-CB (*n* = 3124, 75.1%)	CB (*n* = 1037, 24.9%)	*P*-value
Age	65.4 ± 8.7	62.5 ± 8.6	< 0.01	66.0 ± 8.4	64.0 ± 8.9	< 0.01
Sex (male)	3310 (67.7%)	903 (66.9%)	0.60	2263 (72.4%)	757 (73.0%)	0.76
Race			< 0.01			< 0.01
- NHW	2513 (51.4%)	949 (70.3%)		1392 (44.6%)	468 (45.1%)	
- AA	805 (16.5%)	213 (15.8%)		354 (11.3%)	158 (15.2%)	
- Asian	1572 (32.1%)	188 (13.9%)		1378 (44.1%)	411 (39.6%)	
Smoking status			< 0.01			< 0.01
-Ex-smoker	3188 (65.2%)	602 (44.6%)		2099 (67.2%)	532 (51.3%)	
-Current smoker	1702 (34.8%)	748 (55.4%)		1025 (32.8%)	404 (48.7%)	
Smoking pack-year	48.2 ± 26.0	54.5 ± 28.1	< 0.01	46.5 ± 24.4	50.2 ± 26.5	< 0.01
BMI	26.3 ± 5.8	27.1 ± 6.3	< 0.01	25.9 ± 5.6	25.9 ± 5.8	1.00
Comorbidities						
- DM	681 (13.9%)	174 (12.9%)	0.35	438 (14.0%)	140 (13.5%)	0.71
- HTN	2230 (45.6%)	636 (47.1%)	0.34	1364 (43.7%)	435 (41.9%)	0.35
- Myocardial infarction	313 (6.4%)	103 (7.6%)	0.12	187 (6.0%)	56 (5.4%)	0.54
- Heart failure	206 (4.2%)	57 (4.2%)	1.00	87 (2.8%)	47 (4.5%)	< 0.01
- GERD	1122 (22.9%)	398 (29.5%)	< 0.01	680 (21.8%)	257 (24.8%)	0.049
- Stroke or TIA	201 (5.4%)	64 (5.3%)	0.94	92 (4.4%)	45 (6.3%)	0.049
mMRC	1.6 ± 1.3	2.3 ± 1.4	< 0.01	1.3 ± 1.2	1.9 ± 1.3	< 0.01
Total SGRQ score	31.7 ± 20.8	49.0 ± 21.0	< 0.01	27.7 ± 18.8	43.3 ± 21.6	< 0.01
- Symptom	36.6 ± 22.3	62.9 ± 19.5	< 0.01	34.8 ± 21.4	54.5 ± 22.5	< 0.01
- Activity	46.0 ± 27.8	61.3 ± 25.9	< 0.01	40.6 ± 25.8	54.3 ± 26.5	< 0.01
- Impact	22.0 ± 19.8	37.8 ± 22.2	< 0.01	18.2 ± 17.4	33.6 ± 22.4	< 0.01
CAT score	13.5 ± 7.9	19.6 ± 8.3	< 0.01	9.7 ± 7.0	22.0 ± 7.2	< 0.01
- CAT1 (cough)	1.8 ± 1.3	3.0 ± 1.3	< 0.01	1.4 ± 1.1	3.7 ± 0.8	< 0.01
- CAT2 (sputum)	1.7 ± 1.4	2.8 ± 1.4	< 0.01	1.3 ± 1.1	3.6 ± 0.8	< 0.01
6MWT (m)	381.1 ± 123.0	365.1 ± 117.9	< 0.01	404.6 ± 112.1	381.3 ± 116.6	< 0.01
Depression	406 (15.0%)	140 (22.1%)	< 0.01	348 (13.7%)	202 (24.5%)	< 0.01
Anxiety	371 (14.5%)	114 (18.4%)	0.02	302 (12.5%)	185 (23.8%)	< 0.01
Blood eosinophil count	209.1 ± 202.9	208.0 ± 199.6	0.90	207.7 ± 204.5	211.9 ± 194.2	0.58
GOLD stage			< 0.01			< 0.01
- I (FEV1 ≥ 80%)	853 (17.4%)	136 (10.1%)		610 (19.5%)	107 (10.3%)	
- II (FEV1 50–80%)	2294 (46.9%)	602 (44.6%)		1594 (51.0%)	528 (50.9%)	
- III (FEV1 30–50%)	1223 (25.0%)	430 (31.9%)		759 (24.3%)	327 (31.5%)	
- IV (FEV1 < 30%)	519 (10.6%)	182 (13.5%)		160 (5.1%)	75 (7.2%)	
postBD FEV1 (L)	1.7 ± 0.7	1.6 ± 0.7	< 0.01	1.8 ± 0.7	1.6 ± 0.6	< 0.01
postBD FEV1 (%)	58.8 ± 21.7	53.3 ± 20.3	< 0.01	62.1 ± 20.0	56.4 ± 18.3	< 0.01
postBD FVC (L)	3.2 ± 1.0	3.2 ± 1.0	0.64	3.3 ± 0.9	3.2 ± 0.9	< 0.01
postBD FVC (%)	82.2 ± 19.2	79.9 ± 19.4	< 0.01	84.6 ± 17.9	81.5 ± 17.3	< 0.01
postBD FEV1/FVC	0.52 ± 0.13	0.50 ± 0.13	< 0.01	0.54 ± 0.12	0.51 ± 0.12	< 0.01
DLco	64.8 ± 22.2	62.5 ± 21.0	0.02	65.4 ± 22.1	61.2 ± 21.3	< 0.01
Emphysema on CT	2196 (56.2%)	683 (58.0%)	0.28	1216 (51.7%)	454 (55.5%)	0.07
Medications			< 0.01			< 0.01
- no inhaler	785 (27.8%)	163 (25.5%)		705 (27.8%)	250 (28.0%)	
- LABA or LAMA	542 (19.2%)	101 (15.8%)		494 (19.5%)	157 (17.6%)	
- LABA/LAMA	296 (10.5%)	35 (5.5%)		274 (10.8%)	64 (7.2%)	
- ICS/LABA	395 (14.0%)	98 (15.3%)		358 (14.1%)	137 (15.4%)	
- Triple therapy	808 (28.6%)	242 (37.9%)		707 (27.9%)	284 (31.8%)	
Past exacerbation	1305 (27.0%)	582 (43.3%)	< 0.01	710 (23.1%)	331 (32.3%)	< 0.01
Past severe exacerbation	727 (15.0%)	319 (23.7%)	< 0.01	355 (11.5%)	177 (17.3%)	< 0.01

Data are presented as n (%) or mean ± SD

Demographic data in COPDGene was based on phase I database, except for CAT score and HADS score, which were based on phase II database

All demographic data in KOCOSS was based on the data at the baseline of the study

BMI Body mass index, DM Diabetes mellitus, HTN Hypertension, GERD Gastroesophageal reflux disease, CT Computed tomography, BDI Beck Depression Inventory, BAI Beck Anxiety Inventory, mMRC modified Medical Research Council,, CAT COPD Assessment Test, 6MWT 6-minute walk distance test, ACO Asthma-COPD overlap

LAMA long-acting muscarinic antagonist, LABA long-acting beta2-agonist, ICS inhaled corticosteroids

**Fig. 1:**
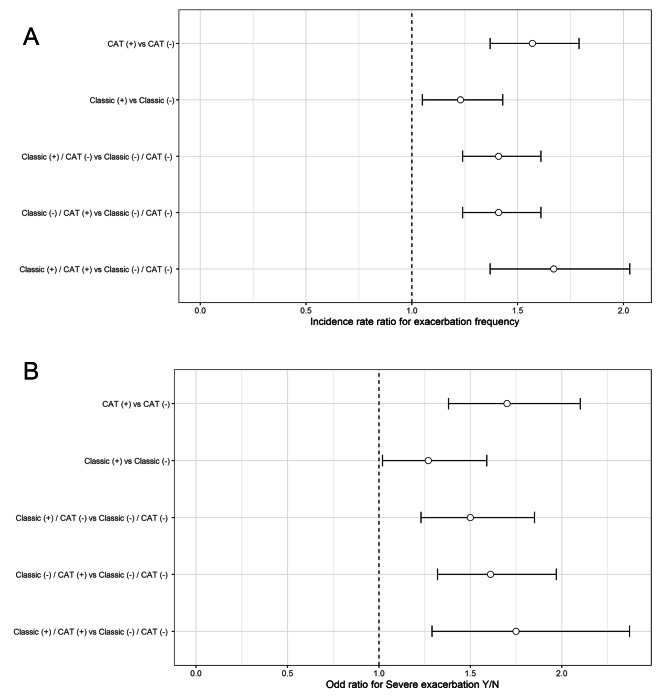
**(A)** Difference in exacerbation frequency between the CB and non-CB groups according to CB definition; **(B)** Difference in severe exacerbation risk between the CB and non-CB groups according to CB definition. Age, sex, smoking status, baseline post-bronchodilator FEV1 (%) and history of exacerbation were adjusted in all analysis

The frequency of exacerbation was significantly higher in the CB than non-CB group, regardless of CB definition (classical definition: incidence rate ratio [IRR] = 1.23, 95% confidence interval [CI], 1.05–1.43; CAT-based definition: IRR = 1.57, 95% CI, 1.37–1.79) (Fig. 1A). Patients with CB had a significantly higher risk of exacerbations compared to the non-CB patients (classical definition: IRR = 1.41, 95% CI, 1.24–1.61; CAT-based definition: IRR = 1.41, 95% CI, 1.24–1.61; both definitions: IRR = 1.67, 95% CI, 1.37–2.03).

The risk of severe exacerbations was significantly higher in CB patients, regardless of CB definition, compared to the non-CB patients (classical definition: OR = 1.27, 95% CI, 1.02–1.59; CAT-based definition: OR = 1.70, 95% CI, 1.38–2.10) (Fig. 1B). CB patients defined by either of the definition had a significantly higher risk of severe exacerbations compared to the non-CB patients defined by both CB definition (classical definition: OR = 1.50, 95% CI, 1.23–1.85; CAT-based definition: OR = 1.61, 95% CI, 1.32–1.97; both definitions: OR = 1.75, 95% CI, 1.29–2.37).

### Differences in clinical characteristics between the non-CB and CB patients in the three racial groups according to CB definition

The proportions of CB patients in the NHW, AA, and Asian groups according to the classical definition were 27.4%, 20.9%, and 10.7%, respectively (Table 3). All three CB patient groups showed severe symptoms and poor quality of life according to the mMRC, SGRQ, and CAT. Poor 6MWT performance was seen in the NHW patients with CB and COPD. FEV1 (%) was poor in the NHW and Asian patients with CB. Exacerbations were more frequent in CB patients in all racial groups, and severe exacerbations were most frequent in the NHW and AA groups.

**Table 3:** Difference of clinical characteristics between CB and non-CB by three different race (classic definition)

	NHW			AA			Asian		
	Non-CB (*n* = 2513, 72.6%)	CB (*n* = 949, 27.4%)	P-value	Non-CB (*n* = 805, 79.1%)	CB (*n* = 213, 20.9%)	*P*-value	Non-CB (*n* = 1572, 89.3%)	CB (*n* = 188, 10.7%)	*P*-value
Age	65.1 ± 8.2	62.4 ± 8.4	< 0.01	58.8 ± 8.3	57.9 ± 7.7	0.14	69.2 ± 7.7	68.2 ± 7.4	0.09
Sex (male)	1352 (53.8%)	596 (62.8%)	< 0.01	432 (53.7%)	124 (58.2%)	0.27	1526 (97.1%)	183 (97.3%)	1.00
Smoking status			< 0.01			< 0.01			0.36
-Ex-smoker	1765 (70.2%)	420 (44.3%)		298 (37.0%)	54 (25.4%)		1125 (71.6%)	128 (68.1%)	
-Current smoker	748 (29.8%)	529 (55.7%)		507 (63.0%)	159 (74.6%)		447 (28.4%)	60 (31.9%)	
Smoking pack-year	53.0 ± 27.2	57.9 ± 28.4	< 0.01	41.6 ± 23.1	44.4 ± 25.1	0.12	44.1 ± 24.0	49.1 ± 26.5	< 0.01
BMI	27.9 ± 5.8	27.9 ± 6.2	0.91	27.9 ± 6.5	27.9 ± 7.3	0.96	23.0 ± 3.4	22.1 ± 3.1	< 0.01
Comorbidities									
- DM	283 (11.3%)	111 (11.7%)	0.76	125 (15.5%)	30 (14.1%)	0.68	273 (17.4%)	33 (17.6%)	1.00
- HTN	1172 (46.7%)	445 (46.9%)	0.93	423 (52.5%)	121 (56.8%)	0.30	635 (40.4%)	70 (37.2%)	0.45
- Myocardial infarction	201 (8.0%)	85 (9.0%)	0.40	44 (5.5%)	15 (7.0%)	0.48	68 (4.3%)	3 (1.6%)	0.11
- Heart failure	115 (4.6%)	39 (4.1%)	0.62	37 (4.6%)	14 (6.6%)	0.32	54 (3.4%)	4 (2.1%)	0.46
- GERD	779 (31.0%)	328 (34.6%)	0.047	150 (18.6%)	48 (22.5%)	0.24	193 (12.3%)	22 (11.7%)	0.91
- Stroke or TIA	145 (5.8%)	52 (5.5%)	0.81	46 (5.7%)	11 (5.2%)	0.90	10 (2.6%)	1 (2.1%)	1.00
mMRC	1.7 ± 1.4	2.3 ± 1.4	< 0.01	1.9 ± 1.5	2.6 ± 1.3	< 0.01	1.68 ± 1.4	2.30 ± 1.4	< 0.01
Total SGRQ score	31.2 ± 21.2	48.4 ± 20.7	< 0.01	36.4 ± 23.6	54.1 ± 21.2	< 0.01	30.0 ± 17.8	46.1 ± 21.5	< 0.01
- Symptom	34.3 ± 23.3	62.9 ± 19.4	< 0.01	38.7 ± 24.9	64.0 ± 21.1	< 0.01	39.3 ± 18.5	61.7 ± 18.4	< 0.01
- Activity	46.6 ± 29.3	61.1 ± 25.7	< 0.01	52.2 ± 30.2	68.0 ± 25.5	< 0.01	41.7 ± 22.8	54.7 ± 25.9	< 0.01
- Impact	21.5 ± 19.3	36.9 ± 21.5	< 0.01	26.6 ± 23.1	43.2 ± 22.9	< 0.01	20.4 ± 18.3	36.3 ± 23.9	< 0.01
CAT score	12.7 ± 7.9	19.0 ± 8.2	< 0.01	15.3 ± 8.7	21.6 ± 7.3	< 0.01	13.8 ± 7.6	20.3 ± 8.7	< 0.01
- CAT1 (cough)	1.8 ± 1.3	3.0 ± 1.3	< 0.01	2.1 ± 1.4	3.0 ± 1.2	< 0.01	1.6 ± 1.3	3.0 ± 1.4	< 0.01
- CAT2 (sputum)	1.5 ± 1.3	2.7 ± 1.3	< 0.01	1.7 ± 1.5	2.7 ± 1.4	< 0.01	1.9 ± 1.4	3.1 ± 1.4	< 0.01
6MWT (m)	393.5 ± 121.8	371.1 ± 119.8	< 0.01	339.8 ± 127.8	324.0 ± 114.5	0.10	383.0 ± 116.2	384.6 ± 98.3	0.86
Depression	123 (8.9%)	85 (19.6%)	< 0.01	43 (10.5%)	13 (13.3%)	0.54	240 (26.1%)	42 (41.2%)	< 0.01
Anxiety	158 (11.5%)	79 (18.2%)	< 0.01	64 (15.6%)	16 (16.3%)	0.98	149 (19.2%)	19 (21.8%)	0.66
GOLD stage			< 0.01			0.04			< 0.01
- I (FEV1 ≥ 80%)	511 (20.3%)	98 (10.3%)		152 (18.9%)	26 (12.2%)		190 (12.1%)	12 (6.4%)	
- II (FEV1 50–80%)	1034 (41.1%)	410 (43.2%)		369 (45.8%)	113 (53.1%)		891 (56.7%)	79 (42.0%)	
- III (FEV1 30–50%)	627 (25.0%)	291 (30.7%)		188 (23.4%)	56 (26.3%)		408 (26.0%)	83 (44.1%)	
- IV (FEV1 < 30%)	341 (13.6%)	150 (15.8%)		96 (11.9%)	18 (8.5%)		82 (5.2%)	14 (7.4%)	
Blood eosinophil count	200.5 ± 144.9	207.0 ± 191.4	0.52	166.3 ± 133.2	170.8 ± 148.2	0.77	231.4 ± 261.7	232.4 ± 240.3	0.96
postBD FEV1 (L)	1.7 ± 0.8	1.6 ± 0.8	< 0.01	1.6 ± 0.7	1.6 ± 0.6	0.66	1.7 ± 0.6	1.5 ± 0.5	< 0.01
postBD FEV1 (%)	58.4 ± 23.5	52.8 ± 20.7	< 0.01	59.5 ± 22.6	57.5 ± 20.4	0.25	59.1 ± 18.0	50.9 ± 16.9	< 0.01
postBD FVC (L)	3.2 ± 1.0	3.2 ± 1.1	0.56	2.8 ± 1.0	2.9 ± 0.9	0.39	3.4 ± 0.8	3.3 ± 0.8	0.52
postBD FVC (%)	82.4 ± 19.9	79.1 ± 19.8	< 0.01	82.9 ± 21.6	82.5 ± 19.7	0.82	81.6 ± 16.4	80.8 ± 16.3	0.53
postBD FEV1/FVC	0.52 ± 0.14	0.50 ± 0.13	< 0.01	0.55 ± 0.12	0.54 ± 0.12	0.21	0.51 ± 0.12	0.45 ± 0.12	< 0.01
DLco	67.9 ± 23.3	65.7 ± 22.0	0.11	57.5 ± 20.6	56.0 ± 19.0	0.57	63.6 ± 21.0	58.2 ± 18.1	< 0.01
Emphysema on CT	1458 (61.5%)	531 (59.7%)	0.39	351 (48.4%)	97 (51.6%)	0.49	387 (47.7%)	55 (55.0%)	0.20
Medications			0.18			0.42			0.03
- no inhaler	307 (32.2%)	97 (26.5%)		106 (35.3%)	26 (30.6%)		372 (23.7%)	40 (21.3%)	
- LABA or LAMA	111 (11.6%)	53 (14.5%)		41 (13.7%)	10 (11.8%)		390 (24.8%)	38 (20.2%)	
- LABA/LAMA	11 (1.2%)	3 (0.8%)		1 (0.3%)	0 (0.0%)		390 (24.8%)	38 (20.2%)	
- ICS/LABA	144 (15.1%)	66 (18.0%)		58 (19.3%)	13 (15.3%)		193 (12.3%)	19 (10.1%)	
- Triple therapy	381 (39.9%)	147 (40.2%)		94 (31.3%)	36 (42.4%)		333 (21.2%)	59 (31.4%)	
Past exacerbation	780 (31.0%)	438 (46.2%)	< 0.01	225 (28.0%)	94 (44.1%)	< 0.01	300 (19.7%)	50 (27.5%)	0.02
Past severe exacerbation	386 (15.4%)	208 (21.9%)	< 0.01	197 (24.5%)	85 (39.9%)	< 0.01	144 (9.5%)	26 (14.3%)	0.06

Data are presented as n (%) or mean ± SD

Demographic data in COPDGene was based on phase I database, except for CAT score and HADS score, which were based on phase II database

All demographic data in KOCOSS was based on the data at the baseline of the study

BMI Body mass index, DM Diabetes mellitus, HTN Hypertension, GERD Gastroesophageal reflux disease, CT Computed tomography, BDI Beck Depression Inventory, BAI Beck Anxiety Inventory, mMRC modified Medical Research Council,, CAT COPD Assessment Test, 6MWT 6-minute walk distance test, ACO Asthma-COPD overlap

LAMA long-acting muscarinic antagonist, LABA long-acting beta2-agonist, ICS inhaled corticosteroids

The proportions of CB patients in the NHW, AA, and Asian groups, diagnosed using the CAT-based definition, were 25.2%, 30.9%, and 23.0%, respectively (Table 4). CB patients in all three racial groups had more severe symptoms and a poorer quality of life according to the mMRC, SGRQ, and CAT. The 6MWT performance was worse in the NHW and AA CB patients. NHW and Asian patients with CB experienced the most symptoms suggestive of depression and anxiety. FEV1(%) was poor in CB patients in all three racial groups. Exacerbations were frequent in all three races, while severe exacerbations were most frequent in the NHW and Asian groups.

**Table 4:** Difference of clinical characteristics between CB and non-CB by three different race (CAT definition)

	NHW			AA			Asian		
	Non-CB (*n* = 1392, 74.8%)	CB (*n* = 468, 25.2%)	*P*-value	Non-CB (*n* = 354, 69.1%)	CB (*n* = 158, 30.9%)	*P*-value	Non-CB (*n* = 1378, 77.0%)	CB (*n* = 411, 23.0%)	*P*-value
Age	64.5 ± 7.7	62.9 ± 8.5	< 0.01	58.6 ± 7.8	57.0 ± 7.3	0.03	69.3 ± 7.6	68.1 ± 7.7	< 0.01
Sex (male)	742 (53.3%)	284 (60.7%)	< 0.01	182 (51.4%)	74 (46.8%)	0.39	1339 (97.2%)	399 (97.1%)	1.00
Smoking status			< 0.01			0.53			< 0.01
-Ex-smoker	970 (69.7%)	234 (50.0%)		110 (31.1%)	44 (27.8%)		1019 (73.9%)	254 (61.8%)	
-Current smoker	422 (30.3%)	234 (50.0%)		244 (68.9%)	114 (72.2%)		359 (26.1%)	157 (38.2%)	
Smoking pack-year	50.1 ± 25.0	56.2 ± 27.2	< 0.01	41.9 ± 21.9	41.5 ± 23.4	0.88	44.0 ± 24.0	46.6 ± 25.4	0.06
BMI	28.3 ± 5.6	28.2 ± 5.8	0.78	28.1 ± 6.6	28.2 ± 6.7	0.88	23.0 ± 3.4	22.5 ± 3.4	< 0.01
Comorbidities									
- DM	141 (10.1%)	54 (11.3%)	0.52	51 (14.4%)	24 (15.2%)	0.92	246 (17.9%)	63 (15.3%)	0.27
- HTN	619 (44.5%)	204 (43.6%)	0.77	180 (50.8%)	86 (54.4%)	0.51	565 (41.0%)	145 (35.3%)	0.04
- Myocardial infarction	108 (7.8%)	31 (6.6%)	0.48	18 (5.1%)	12 (7.6%)	0.36	61 (4.4%)	13 (3.2%)	0.32
- Heart failure	35 (2.5%)	23 (4.9%)	0.02	6 (1.7%)	10 (6.3%)	0.01	46 (3.3%)	14 (3.4%)	1.00
- GERD	450 (32.4%)	168 (35.9%)	0.18	64 (18.1%)	36 (22.8%)	0.26	166 (12.0%)	53 (12.9%)	0.71
- Stroke or TIA	71 (5.1%)	33 (7.1%)	0.14	12 (3.4%)	10 (6.3%)	0.20	9 (2.5%)	2 (2.3%)	1.00
mMRC	1.4 ± 1.4	1.9 ± 1.4	< 0.01	1.7 ± 1.5	1.5 ± 1.4	< 0.01	1.23 ± 0.84	1.62 ± 0.95	< 0.01
Total SGRQ score	26.5 ± 19.9	40.3 ± 21.1	< 0.01	33.5 ± 23.1	45.3 ± 23.0	< 0.01	27.4 ± 15.8	46.0 ± 21.2	< 0.01
- Symptom	32.2 ± 23.3	51.8 ± 24.5	< 0.01	37.8 ± 25.9	52.4 ± 25.0	< 0.01	36.7 ± 17.3	58.4 ± 18.3	< 0.01
- Activity	39.5 ± 27.9	52.5 ± 26.8	< 0.01	47.9 ± 30.1	59.7 ± 27.2	< 0.01	39.7 ± 21.8	54.2 ± 25.8	< 0.01
- Impact	17.4 ± 17.6	30.0 ± 20.5	< 0.01	23.9 ± 21.8	34.9 ± 23.7	< 0.01	17.5 ± 15.6	37.4 ± 23.4	< 0.01
CAT score	11.5 ± 7.0	22.2 ± 6.9	< 0.01	13.3 ± 7.9	23.7 ± 6.4	< 0.01	12.0 ± 6.4	22.7 ± 7.3	< 0.01
- CAT1 (cough)	1.6 ± 1.1	3.8 ± 0.8	< 0.01	1.7 ± 1.2	3.6 ± 0.8	< 0.01	1.2 ± 1.0	3.6 ± 0.8	< 0.01
- CAT2 (sputum)	1.2 ± 1.0	3.6 ± 0.7	< 0.01	1.1 ± 1.1	3.6 ± 0.8	< 0.01	1.5 ± 1.1	3.7 ± 0.8	< 0.01
6MWT (m)	428.1 ± 104.8	399.1 ± 112.8	< 0.01	364.8 ± 110.2	341.4 ± 128.9	< 0.01	386.1 ± 115.1	375.2 ± 110.2	0.14
Depression	111 (8.2%)	97 (21.3%)	< 0.01	36 (10.2%)	20 (12.7%)	0.050	201 (24.3%)	85 (39.9%)	< 0.01
Anxiety	147 (10.8%)	90 (19.8%)	< 0.01	40 (11.4%)	40 (25.5%)	0.50	115 (16.2%)	55 (33.1%)	< 0.01
Blood eosinophil count	197.4 ± 145.7	215.9 ± 186.8	0.06	168.2 ± 134.3	165.5 ± 140.6	< 0.01	231.5 ± 268.4	227.9 ± 220.1	0.80
GOLD stage			< 0.01			0.01			< 0.01
- I (FEV1 ≥ 80%)	354 (25.4%)	59 (12.6%)		78 (22.0%)	18 (11.4%)		178 (12.9%)	30 (7.3%)	
- II (FEV1 50–80%)	636 (45.7%)	236 (50.4%)		187 (52.8%)	84 (53.2%)		771 (56.0%)	208 (50.6%)	
- III (FEV1 30–50%)	314 (22.6%)	144 (30.8%)		76 (21.5%)	47 (29.7%)		369 (26.8%)	136 (33.1%)	
- IV (FEV1 < 30%)	88 (6.3%)	29 (6.2%)		13 (3.7%)	9 (5.7%)		59 (4.3%)	37 (9.0%)	
postBD FEV1 (L)	1.9 ± 0.8	1.7 ± 0.7	< 0.01	1.7 ± 0.7	1.5 ± 0.6	< 0.01	1.7 ± 0.6	1.6 ± 0.6	< 0.01
postBD FEV1 (%)	63.9 ± 21.7	57.9 ± 18.6	< 0.01	64.7 ± 19.5	59.2 ± 18.8	< 0.01	59.6 ± 18.0	53.6 ± 17.6	< 0.01
postBD FVC (L)	3.4 ± 1.0	3.3 ± 1.0	0.11	3.0 ± 0.9	2.7 ± 0.8	< 0.01	3.4 ± 0.8	3.3 ± 0.8	0.051
postBD FVC (%)	86.3 ± 18.8	82.5 ± 17.7	< 0.01	87.8 ± 19.0	81.1 ± 17.8	< 0.01	82.1 ± 16.4	80.4 ± 16.5	0.07
postBD FEV1/FVC	0.55 ± 0.12	0.53 ± 0.12	< 0.01	0.57 ± 0.10	0.57 ± 0.10	0.51	0.51 ± 0.11	0.48 ± 0.13	< 0.01
DLco	68.4 ± 23.2	64.1 ± 21.9	< 0.01	58.4 ± 20.4	54.2 ± 19.6	0.06	63.8 ± 20.7	60.4 ± 20.5	< 0.01
Emphysema on CT	738 (55.6%)	270 (60.1%)	0.10	152 (46.3%)	64 (44.8%)	0.83	326 (46.8%)	120 (53.1%)	0.12
Medications			0.32			0.78			0.01
- no inhaler	304 (33.6%)	100 (27.9%)		93 (36.5%)	39 (32.0%)		308 (22.4%)	111 (27.0%)	
- LABA or LAMA	112 (12.4%)	52 (14.5%)		31 (12.2%)	19 (15.6%)		351 (25.5%)	86 (20.9%)	
- LABA/LAMA	9 (1.0%)	5 (1.4%)		1 (0.4%)	0 (0.0%)		264 (19.2%)	59 (14.4%)	
- ICS/LABA	145 (16.0%)	65 (18.1%)		48 (18.8%)	23 (18.9%)		165 (12.0%)	49 (11.9%)	
- Triple therapy	335 (37.0%)	137 (38.2%)		82 (32.2%)	41 (33.6%)		290 (21.0%)	106 (25.8%)	
Past exacerbation	372 (26.7%)	174 (37.2%)	< 0.01	85 (24.0%)	57 (36.1%)	< 0.01	253 (19.0%)	100 (25.0%)	0.01
Past severe exacerbation	156 (11.2%)	78 (16.7%)	< 0.01	80 (22.6%)	47 (29.7%)	0.11	119 (8.9%)	52 (13.0%)	0.02

Data are presented as n (%) or mean ± SD

Demographic data in COPDGene was based on phase I database, except for CAT score and HADS score, which were based on phase II database

All demographic data in KOCOSS was based on the data at the baseline of the study

BMI Body mass index, DM Diabetes mellitus, HTN Hypertension, GERD Gastroesophageal reflux disease, CT Computed tomography, BDI Beck Depression Inventory, BAI Beck Anxiety Inventory, mMRC modified Medical Research Council,, CAT COPD Assessment Test, 6MWT 6-minute walk distance test, ACO Asthma-COPD overlap

LAMA long-acting muscarinic antagonist, LABA long-acting beta2-agonist, ICS inhaled corticosteroids

**Fig. 2:**
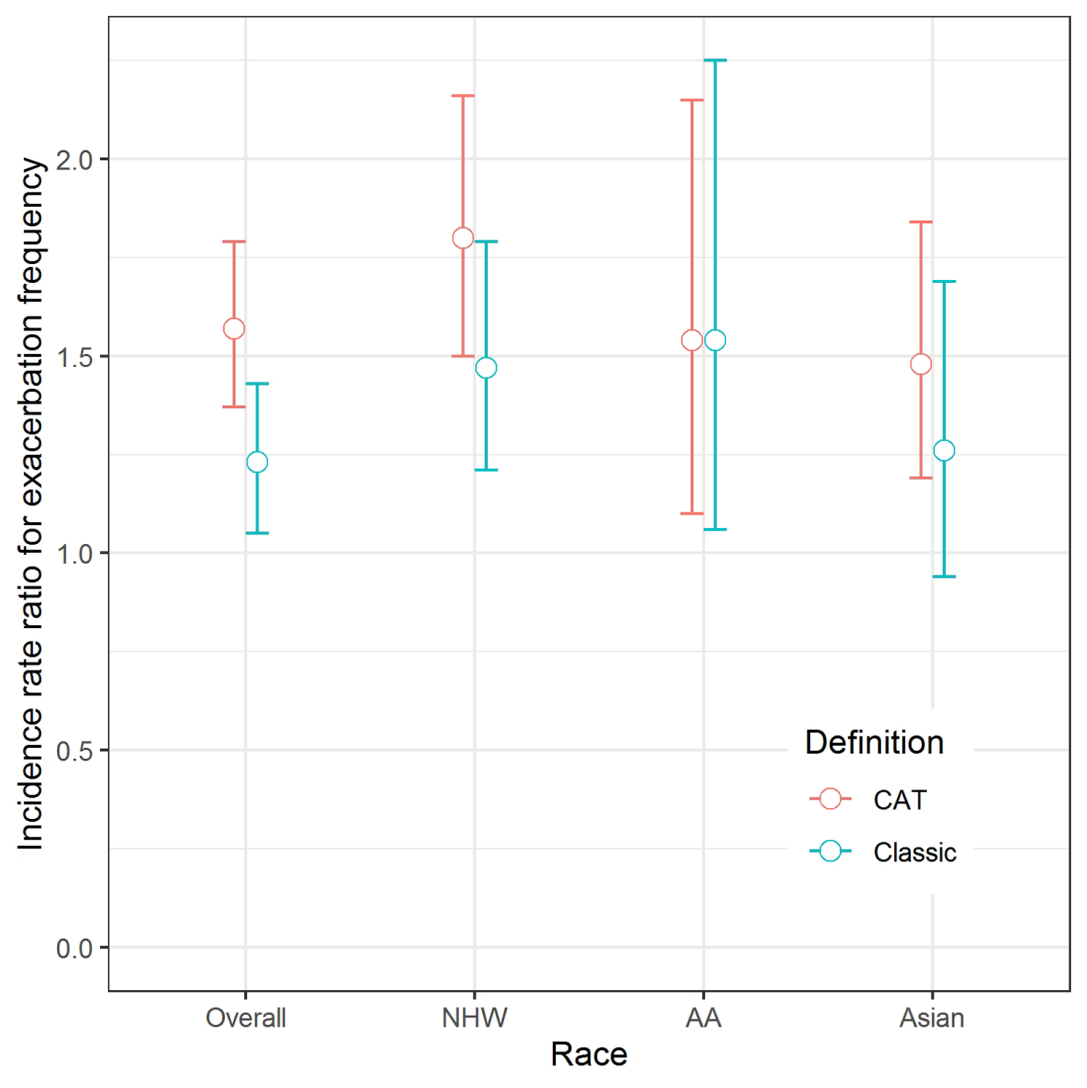
Difference in exacerbation frequency between the CB and non-CB groups according to race and CB definition. Age, sex, smoking status, baseline post-bronchodilator FEV1 (%) and history of exacerbation were adjusted in all analysis

As shown in Fig. 2, the risk of future exacerbations was significantly higher in classically defined CB patients than non-CB patients in the NHW and AA groups, but not in those in the Asian group (overall: IRR = 1.23, 95% CI, 1.05–1.43; NHW: IRR = 1.47, 95% CI, 1.21–1.79; AA: IRR = 1.54, 95% CI, 1.06–2.25; Asian: IRR = 1.26, 95% CI, 0.94–1.69). The risk of future exacerbations was significantly higher for the CAT-defined CB patients than non-CB patients in all racial groups (overall: IRR = 1.57, 95% CI, 1.37–1.79; NHW: IRR = 1.80, 95% CI: 1.50–2.16; AA: IRR = 1.54, 95% CI, 1.10–2.15; Asian: IRR = 1.48, 95% CI, 1.19–1.84).

## Discussion

In this study, we merged the databases of the COPDGene and KOCOSS studies, which were both large nationwide prospective cohort studies, and compared clinical characteristics among three racial groups of CB and non-CB patients. CB patients were more likely to be current smokers, had more severe symptoms, lower exercise capacity, decreased lung function, and more exacerbations than non-CB patients in all three racial groups. Although some discrepancies in clinical characteristics were observed when applying the two different CB definitions, for most of the characteristics the results were comparable. In the prospective 1-year exacerbation analysis, CB patients had an increased risk of future exacerbations, except for Asian patients with CB (when using the classical definition). Risk of CB prevalence was highest in NHW followed by AA, and Asian showed lowest risk of CB prevalence.

There have been limited data on different prevalence of CB phenotype between races. In our study, we compared risk of CB prevalence based on two large high quality cohort database and revealed highest risk of CB prevalence in NHW and lowest in Asian population. A previous study have analyzed different prevalence of cough and sputum in COPDGene and KOCOSS database [22], but we further analyzed with logistic regression model to adjust covariate factors including age, sex, smoking history, lung function and past exacerbation history. In a retrospective cross-sectional study, whites were more likely to be COPD than blacks or Asians, which may imply different susceptibility of risk factors (i.e. tobacco smoking) to airway pathogenesis between races [23]. Genetic and pathophysiologic differences between races may be associated with these differences, and further studies are required.

Previous studies reported that the presence of CB was associated with a poor clinical outcome, which corresponded with our study. However, its clinical significance has not previously been compared among racial groups. The clinical features of COPD differ among racial groups. Park et al. analyzed the COPDGene and KOCOSS databases, as in our study, and showed that NHW patients had more CB symptoms, AA patients complained more of dyspnea and poor exercise capacity, and Asians had a lower BMI and more comorbidities, except diabetes and exacerbations [22]. Jo et al. analyzed ACO patients in the same database and showed that the prevalence of ACO was highest in Asians, while the risk of exacerbation was higher in NHW patients and Asians than AA patients [24]. These differences could be due to genetic and socioeconomic factors. As there are differences in clinical features between races, whether the clinical significance of CB is different in different racial groups is difficult to determine. In this study, the CB phenotype was consistently associated with a poor clinical outcome.

One of the most important limitations of the classic definition is recall bias, where patients must remember 2 years of CB symptoms [9, 13]. To overcome this limitation, our group previously proposed a CAT-based definition based on the prevalence of CB in the general COPD population [16]. We validated this definition based on CT parameters and the clinical manifestations of CB, and demonstrated that it can be used to predict the risk of future exacerbations [9, 13, 16]. However, there are limitations to the CAT definition; it has only been validated in Asian patients, such that validation in other racial groups is needed. In this study, we showed that the CAT-based definition may also be used in other racial groups. CAT-defined CB was associated with poor outcomes and the risk of future exacerbations. When using the classical definition, statistical significance was not met with respect to predicting future exacerbations in the Asian group, although that was not the case when using the CAT definition. Thus, the CAT-based definition may be superior for predicting the risk of future exacerbations compared to the classic definition. Additionally, the CAT definition can easily be applied in clinical practice.

While the classic definition of CB is prone to recall bias, it does have the advantages of assessing symptom presence over a two-year period. In contrast, the CAT-based definition relies predominantly on symptom reports from a relatively brief prior to questionnaire administration. To address this limitation, the use of electronic symptom diaries, as employed in large randomized controlled trials could serve as an alternative [25]. Such diaries allow for the objective and timely collection of CB symptoms over days, weeks, and months, providing a more dynamic assessment of the condition.

Exacerbation is the most important risk factor for poor HrQOL [26], accelerated decline in lung function [27, 28], future exacerbations [29], comorbidities (ischemic heart disease, pneumonia, and diabetes) [30] and mortality [31] in COPD patients. Thus, one of the most important goals when managing COPD patients is to identify those at risk of exacerbations and prevent them from occurring. A previous study using the COPDGene database showed that the SGRQ-based definition better predicted the risk of future exacerbations [15]. Our previous study also showed that CAT-defined CB patients are at risk of future exacerbations [9, 13]. In this study, we demonstrated that the classical and CAT-based definitions of CB predict future exacerbations, including severe ones. When using the classical definition, statistical significance was not met with respect to predicting future exacerbations in the Asian group, although that was not the case when using the CAT definition. Thus, the CAT-based definition may be superior for predicting the risk of future exacerbations compared to the classic definition.

The use of specific medications may have influenced the outcomes. Given that the CB phenotype is associated with more severe disease, the use of triple therapy was more prevalent among CB patients. Despite more frequent use of triple therapy and ICS/LABA in the CB group compared to the non-CB group, these patients still exhibited a higher risk of future exacerbations. This indicates the importance of the CB phenotype as a critical prognostic factor for exacerbations. Furthermore, among racial groups, NHW demonstrated higher utilization of triple therapy and also showed an increased risk of developing the CB phenotype and subsequent exacerbations. This suggests that, despite the use of triple therapy, CB is still associated with an increased risk of exacerbations. Unfortunately, the use of LABA/LAMA was minimal in the COPDGene study, as it was based on Phase I data, a time when LABA/LAMA therapies were not widely used.

Some limitations of our study should be discussed. First, there were discrepancies in the exacerbation “timeline” between the two databases. For the KOCOSS cohort, we analyzed 1-year prospective data for exacerbations, and adjusted for the 1-year exacerbation history recorded at baseline. However, for the analysis of the COPDGene cohort, we analyzed 1-year exacerbation data in phase II, with adjustment for the 1-year exacerbation history recorded in phase I. Thus, the temporal gap between the outcome variable and covariates was greater in the COPDGene study. Moreover, in the COPDGene cohort, the CB phenotype defined by the CAT definition was based on Phase II data, while the classic definition was based on Phase I data. Second, most patients included in the KOCOSS cohort were male, and the analysis of the baseline characteristics of the CB patients may have been affected by sex differences in COPD prevalence. In particular, most CB patients in other racial groups are male [8], and the prevalence of CB in the Asians may have been overestimated in our study. To overcome this limitation, we conducted further analyses on the varying characteristics of CB across different races among male patients (Table S2-S6, Fig S2-S4) and in an age- and sex-matched population using propensity score matching (Table S7-S9); we found results similar to those presented in the main findings. Third, CAT and HADS score in COPDGene study were based on phase II visit, because those scores were not collected at phase I visit. Consequently, the CAT-defined CB is based on the phase II visit, and we have assumed that the CB status remained unchanged from the baseline. Fourth, the presence of bronchiectasis in patients with COPD can alter clinical outcomes significantly, especially resulting in increased exacerbations [32, 33]. Unfortunately, our current analysis is limited by the absence of bronchiectasis data within the COPDGene dataset. However, emerging methodologies using AI to identify bronchiectasis within the COPDGene study warrant further research [34]. Finally, the two databases used different scoring systems to evaluate psychological status. Thus, the results should not be used as a basis to compare mental health between Asians and the other two races. Despite these limitations, our study is the first to demonstrate the clinical significance of the CB phenotype in three racial groups. We showed that the CAT-based definition is a useful alternative definition and may better predict future exacerbation risk.

## Conclusions

The risk of CB prevalence is highest in NHW, followed by AA, and least in Asian COPD patients. CB phenotype was associated with a poor clinical outcome, including more severe symptoms, low exercise capacity, poor mental health, and deterioration of lung function, regardless of race (NWH, AA or Asian) or CB definition (classical or CAT-based). Additionally, CB phenotype was an independent risk factor for future exacerbations, including severe ones, regardless of CB definition. However, the risk of exacerbations in Asian patients was only associated with CAT-defined CB. These results suggest that the CAT-based definition could serve as an alternative CB definition, and may be superior for assessing the risk of future exacerbations.

### Electronic supplementary material

Below is the link to the electronic supplementary material.

Supplementary Material 1

## Data Availability

The datasets supporting the conclusions of this article are available from the corresponding author on reasonable request.
