# Supplementary material for: Clinical significance of chronic bronchitis in different racial groups
Source: BMC Pulm Med. 2024 Jun 17;24:282. doi: 10.1186/s12890-024-03100-y (PMC11184853; doi:10.1186/s12890-024-03100-y)

Table S1. Risks of CB prevalence according to 3 different races

|  | Classic definition | | | CAT definition | | |
| --- | --- | --- | --- | --- | --- | --- |
|  | OR | 95%CI | p-value | OR | 95%CI | p-value |
| Race |  |  |  |  |  |  |
| Asian | 1 (reference) |  |  | 1 (reference) |  |  |
| AA | 1.46 | 1.15-1.86 | <0.01 | 1.07 | 0.83-1.39 | 0.60 |
| NHW | 3.18 | 2.64-3.84 | <0.01 | 1.23 | 1.02-1.47 | 0.03 |

Adjusted covariates include age, sex, smoking history, post-bronchodilator FEV_1_, past exacerbation history

AA African American, NHW Non-Hispanic White, CAT COPD Assessment Test

Table S2. Baseline characteristics of COPDGene and KOCOSS data in male patients

|  | NHW  (n=1,948) | AA  (n=556) | Asian  (n=1,742) | P-value |
| --- | --- | --- | --- | --- |
| Age | 64.6±8.3 | 58.3±8.0 | 69.1±7.7 | <0.01 |
| Smoking status |  |  |  | <0.01 |
| -Ex-smoker | 1219 (62.6%) | 167 (30.0%) | 1244 (71.4%) |  |
| -Current smoker | 729 (37.4%) | 389 (70.0%) | 498 (28.6%) |  |
| Smoking pack-year | 58.6±29.8 | 45.1±24.5 | 45.0±24.4 | <0.01 |
| BMI | 28.2±5.5 | 26.7±5.8 | 22.9±3.4 | <0.01 |
| Comorbidities |  |  |  |  |
| - DM | 267 (13.7%) | 77 (13.8%) | 303 (17.4%) | <0.01 |
| - HTN | 943 (48.4%) | 267 (48.0%) | 693 (39.8%) | <0.01 |
| - Myocardial infarction | 214 (11.0%) | 32 (5.8%) | 71 (4.1%) | <0.01 |
| - Heart failure | 93 (4.8%) | 30 (5.4%) | 59 (3.4%) | 0.04 |
| - GERD | 552 (28.4%) | 87 (15.6%) | 213 (12.2%) | <0.01 |
| - Stroke or TIA | 123 (6.3%) | 22 (4.0%) | 10 (2.3%) | <0.01 |
| mMRC | 1.8±1.5 | 1.8±1.5 | 1.3±0.9 | <0.01 |
| Total SGRQ score | 35.4±22.3 | 38.2±24.7 | 31.7±18.9 | <0.01 |
| - Symptom | 43.1±25.4 | 43.6±26.7 | 41.7±19.8 | 0.12 |
| - Activity | 48.8±29.1 | 51.8±30.2 | 43.0±23.6 | <0.01 |
| - Impact | 25.4±20.9 | 28.9±24.6 | 22.1±19.5 | <0.01 |
| CAT score | 14.2±8.5 | 15.8±8.8 | 14.4±8.0 | 0.02 |
| - CAT1 (cough) | 2.2±1.4 | 2.2±1.4 | 1.7±1.4 | <0.01 |
| - CAT2 (sputum) | 1.9±1.4 | 1.8±1.5 | 2.0±1.4 | 0.04 |
| CB (classic definition) | 596 (30.6%) | 124 (22.3%) | 183 (10.7%) | <0.01 |
| CB (CAT definition) | 284 (27.7%) | 74 (28.9%) | 399 (23.0%) | <0.01 |
| 6MWT (m) | 397.1±124. | 4354.9±125. | 0384.0±114.9 | <0.01 |
| Depression | 111 (11.1%) | 30 (11.9%) | 276 (27.3%) | <0.01 |
| Anxiety | 104 (10.4%) | 32 (12.6%) | 165 (19.3%) | <0.01 |
| Blood eosinophil count | 216.2±174.1 | 175.6±151.8 | 232.4±259.3 | <0.01 |
| GOLD stage |  |  |  | <0.01 |
| - I (FEV1 ≥80%) | 357 (18.3%) | 96 (17.3%) | 200 (11.5%) |  |
| - II (FEV1 50-80%) | 775 (39.8%) | 255 (45.9%) | 949 (54.5%) |  |
| - III (FEV1 30-50%) | 530 (27.2%) | 135 (24.3%) | 498 (28.6%) |  |
| - IV (FEV1 <30%) | 286 (14.7%) | 70 (12.6%) | 94 (5.4%) |  |
| postBD FEV1 (L) | 1.9±0.9 | 1.8±0.8 | 1.7±0.6 | <0.01 |
| postBD FEV1 (%) | 56.8±23.3 | 58.6±22.3 | 58.1±18.1 | 0.08 |
| postBD FVC (L) | 3.6±1.0 | 3.3±0.9 | 3.4±0.8 | <0.01 |
| postBD FVC (%) | 81.3±20.0 | 82.2±20.5 | 81.4±16.4 | 0.60 |
| postBD FEV1/FVC | 0.51±0.14 | 0.54±0.13 | 0.50±0.12 | <0.01 |
| DLco | 68.7±23.7 | 56.7±20.3 | 62.9±20.7 | <0.01 |
| Emphysema on CT | 1191 (64.6%) | 268 (53.3%) | 438 (48.9%) | <0.01 |
| Medications |  |  |  | <0.01 |
| - no inhaler | 236 (33.4%) | 67 (37.4%) | 411 (23.6%) |  |
| - LABA or LAMA | 87 (12.3%) | 24 (13.4%) | 428 (24.6%) |  |
| - LABA/LAMA | 9 (1.3%) | 0 (0.0%) | 319 (18.3%) |  |
| - ICS/LABA | 102 (14.4%) | 31 (17.3%) | 201 (11.5%) |  |
| - ICS/LABA/LAMA | 273 (38.6%) | 57 (31.8%) | 383 (22.0%) |  |
| Past exacerbation | 612 (31.4%) | 156 (28.1%) | 341 (20.2%) | <0.01 |
| Past severe exacerbation | 331 (17.0%) | 142 (25.5%) | 163 (9.7%) | <0.01 |

Data are presented as n (%) or mean ± SD

Demographic data in COPDGene was based on phase I database, except for CAT score and HADS score, which were based on phase II database.

All demographic data in KOCOSS was based on the data at the baseline of the study.

BMI Body mass index, DM Diabetes mellitus, HTN Hypertension, GERD Gastroesophageal reflux disease, CT Computed tomography, BDI Beck Depression Inventory, BAI Beck Anxiety Inventory, mMRC modified Medical Research Council, , CAT COPD Assessment Test, 6MWT 6-minute walk distance test, ACO Asthma-COPD overlap

LAMA long-acting muscarinic antagonist, LABA long-acting beta2-agonist, ICS inhaled corticosteroids

Table S3. Risks of CB prevalence according to 3 different races in male patients

|  | Classic definition | | | CAT definition | | |
| --- | --- | --- | --- | --- | --- | --- |
|  | OR | 95%CI | p-value | OR | 95%CI | p-value |
| Race |  |  |  |  |  |  |
| Asian | 1 (reference) |  |  | 1 (reference) |  |  |
| AA | 1.39 | 1.05-1.84 | 0.02 | 0.88 | 0.64-1.23 | 0.46 |
| NHW | 3.31 | 2.73-4.01 | <0.01 | 1.34 | 1.11-1.63 | <0.01 |

Adjusted covariates include age, sex, smoking history, post-bronchodilator FEV_1_, past exacerbation history

AA African American, NHW Non-Hispanic White, CAT COPD Assessment Test

Table S4. Difference of clinical characteristics between CB and non-CB according to 2 different definitions in male patients

|  | Classic definition | | | CAT definition | | |
| --- | --- | --- | --- | --- | --- | --- |
|  | Non-CB  (n=3310, 78.6%) | CB  (n=903, 21.4%) | P-value | Non-CB  (n=2263, 74.9%) | CB  (n=757, 25.1%) | P-value |
| Age | 66.2±8.7 | 63.4±8.6 | <0.01 | 66.9±8.3 | 65.3±8.7 | <0.01 |
| Race |  |  | <0.01 |  |  | <0.01 |
| - NHW | 1352 (40.8%) | 596 (66.0%) |  | 742 (32.8%) | 284 (37.5%) |  |
| - AA | 432 (13.1%) | 124 (13.7%) |  | 182 (8.0%) | 74 (9.8%) |  |
| - Asian | 1526 (46.1%) | 183 (20.3%) |  | 1339 (59.2%) | 399 (52.7%) |  |
| Smoking status |  |  | <0.01 |  |  | <0.01 |
| -Ex-smoker | 2174 (65.7%) | 434 (48.1%) |  | 1561 (69.0%) | 419 (55.4%) |  |
| -Current smoker | 1136 (34.3%) | 469 (51.9%) |  | 702 (31.0%) | 338 (44.6%) |  |
| Smoking pack-year | 49.7±27.1 | 57.4±29.8 | <0.01 | 47.3±25.4 | 51.6±27.3 | <0.01 |
| BMI | 25.6±5.3 | 26.6±5.8 | <0.01 | 25.2±5.0 | 25.1±5.2 | 0.64 |
| Comorbidities |  |  |  |  |  |  |
| - DM | 521 (15.7%) | 122 (13.5%) | 0.11 | 363 (16.0%) | 106 (14.0%) | 0.20 |
| - HTN | 1475 (44.6%) | 421 (46.6%) | 0.29 | 989 (43.7%) | 292 (38.6%) | 0.02 |
| - Myocardial infarction | 237 (7.2%) | 77 (8.5%) | 0.19 | 144 (6.4%) | 44 (5.8%) | 0.65 |
| - Heart failure | 143 (4.3%) | 37 (4.1%) | 0.84 | 70 (3.1%) | 34 (4.5%) | 0.09 |
| - GERD | 633 (19.1%) | 215 (23%) | <0.01 | 407 (18.0%) | 161 (21.3%) | 0.052 |
| - Stroke or TIA | 112 (5.2%) | 43 (5.6%) | 0.69 | 58 (4.6%) | 22 (5.0%) | 0.82 |
| mMRC | 1.4±1.2 | 2.1±1.4 | <0.01 | 1.2±1.1 | 1.8±1.2 | <0.01 |
| Total SGRQ score | 30.6±20.0 | 47.6±21.4 | <0.01 | 27.1±18.0 | 43.4±21.3 | <0.01 |
| - Symptom | 37.2±21.5 | 62.4±19.9 | <0.01 | 35.4±20.4 | 55.5±21.5 | <0.01 |
| - Activity | 43.6±26.8 | 58.7±26.2 | <0.01 | 39.1±24.7 | 53.5±26.3 | <0.01 |
| - Impact | 21.2±19.3 | 36.8±22.6 | <0.01 | 17.8±16.9 | 34.0±22.4 | <0.01 |
| CAT score | 13.5±7.8 | 19.3±8.5 | <0.01 | 11.8±6.7 | 22.6±7.1 | <0.01 |
| - CAT1 (cough) | 1.7±1.3 | 3.0±1.4 | <0.01 | 1.3±1.0 | 3.7±0.8 | <0.01 |
| - CAT2 (sputum) | 1.8±1.4 | 2.8±1.4 | <0.01 | 1.4±1.1 | 3.6±0.8 | <0.01 |
| 6MWT (m) | 389.2±123.6 | 376.7±117.0 | <0.01 | 408.9±115.7 | 383.8±114.5 | <0.01 |
| Depression | 319 (17.5%) | 94 (22.3%) | 0.02 | 270 (15.8%) | 147 (26.4%) | <0.01 |
| Anxiety | 231 (13.7%) | 68 (16.7%) | 0.01 | 185 (11.6%) | 116 (22.8%) | <0.01 |
| Blood eosinophil count | 221.3±224.1 | 222.7±222.9 | 0.90 | 220.9±226.8 | 222.1±212.5 | 0.90 |
| GOLD stage |  |  | <0.01 |  |  | <0.01 |
| - I (FEV1 ≥80%) | 554 (16.7%) | 91 (10.1%) |  | 414 (18.3%) | 64 (8.5%) |  |
| - II (FEV1 50-80%) | 1588 (48.0%) | 380 (42.1%) |  | 1168 (51.6%) | 367 (48.5%) |  |
| - III (FEV1 30-50%) | 843 (25.5%) | 306 (33.9%) |  | 567 (25.1%) | 259 (34.2%) |  |
| - IV (FEV1 <30%) | 324 (9.8%) | 126 (14.0%) |  | 113 (5.0%) | 67 (8.9%) |  |
| postBD FEV1 (L) | 1.8±0.8 | 1.7±0.8 | <0.01 | 1.9±0.7 | 1.7±0.7 | <0.01 |
| postBD FEV1 (%) | 58.9±21.2 | 52.7±20.3 | <0.01 | 61.6±19.7 | 54.5±18.4 | <0.01 |
| postBD FVC (L) | 3.5±0.9 | 3.5±1.0 | 0.97 | 3.6±0.9 | 3.4±0.9 | <0.01 |
| postBD FVC (%) | 81.8±18.5 | 80.0±19.4 | 0.01 | 83.7±17.3 | 80.4±17.3 | <0.01 |
| postBD FEV1/FVC | 0.52±0.13 | 0.49±0.13 | <0.01 | 0.53±0.12 | 0.50±0.12 | <0.01 |
| DLco | 65.0±22.2 | 62.1±21.4 | 0.01 | 65.7±22.1 | 60.8±21.4 | <0.01 |
| Emphysema on CT | 1406 (57.2%) | 487 (63.1%) | <0.01 | 844 (54.0%) | 341 (61.1%) | <0.01 |
| Medications |  |  | <0.01 |  |  | <0.01 |
| - no inhaler | 587 (27.3%) | 119 (26.7%) |  | 526 (27.1%) | 187 (28.2%) |  |
| - LABA or LAMA | 457 (21.3%) | 72 (16.1%) |  | 418 (21.5%) | 120 (18.1%) |  |
| - LABA/LAMA | 285 (13.3%) | 34 (7.6%) |  | 267 (13.7%) | 59 (8.9%) |  |
| - ICS/LABA | 268 (12.5%) | 64 (14.3%) |  | 240 (12.4%) | 94 (14.2%) |  |
| - Triple therapy | 552 (25.7%) | 157 (35.2%) |  | 491 (25.3%) | 204 (30.7%) |  |
| Past exacerbation | 765 (23.5%) | 341 (38.0%) | <0.01 | 452 (20.4%) | 223 (29.9%) | <0.01 |
| Past severe exacerbation | 434 (13.3%) | 201 (22.4%) | <0.01 | 227 (10.2%) | 130 (17.4%) | <0.01 |

Data are presented as n (%) or mean ± SD

Demographic data in COPDGene was based on phase I database, except for CAT score and HADS score, which were based on phase II database.

All demographic data in KOCOSS was based on the data at the baseline of the study.

BMI Body mass index, DM Diabetes mellitus, HTN Hypertension, GERD Gastroesophageal reflux disease, CT Computed tomography, BDI Beck Depression Inventory, BAI Beck Anxiety Inventory, mMRC modified Medical Research Council, , CAT COPD Assessment Test, 6MWT 6-minute walk distance test, ACO Asthma-COPD overlap

LAMA long-acting muscarinic antagonist, LABA long-acting beta2-agonist, ICS inhaled corticosteroids

Table S5. Difference of clinical characteristics between CB and non-CB by three different race (classic definition) in male patients

|  | NHW | | | AA | | | Asian | | |
| --- | --- | --- | --- | --- | --- | --- | --- | --- | --- |
|  | Non-CB  (n=1352, 69.4%) | CB  (n=596, 31.6%) | P-value | Non-CB  (n=432, 77.7%) | CB  (n=124, 22.3%) | P-value | Non-CB  (n=1526, 89.3%) | CB  (n=183, 10.7%) | P-value |
| Age | 65.2±8.1 | 63.1±8.5 | <0.01 | 58.4±8.1 | 58.0±7.6 | 0.62 | 69.2±7.7 | 68.1±7.3 | 0.08 |
| Smoking status |  |  | <0.01 |  |  | 0.052 |  |  | 0.56 |
| -Ex-smoker | 940 (69.5%) | 279 (46.8%) |  | 139 (32.2%) | 28 (22.6%) |  | 1095 (71.8%) | 127 (69.4%) |  |
| -Current smoker | 412 (30.5%) | 317 (53.2%) |  | 293 (67.8%) | 96 (77.4%) |  | 431 (28.2%) | 56 (30.6%) |  |
| Smoking pack-year | 57.4±29.5 | 61.3±30.4 | <0.01 | 43.7±23.3 | 50.1±27.8 | 0.02 | 44.5±24.1 | 49.3±26.7 | 0.01 |
| BMI | 28.3±5.4 | 28.0±5.7 | 0.32 | 26.7±5.6 | 26.6±6.2 | 0.85 | 23.0±3.4 | 22.2±3.1 | <0.01 |
| Comorbidities |  |  |  |  |  |  |  |  |  |
| - DM | 194 (14.3%) | 73 (12.2%) | 0.24 | 60 (13.9%) | 17 (13.7%) | 1.00 | 267 (17.5^) | 32 (17.5%) | 1.00 |
| - HTN | 657 (48.6%) | 286 (48.0%) | 0.83 | 198 (45.8%) | 69 (55.6%) | 0.07 | 620 (40.6%) | 66 (36.1%) | 0.27 |
| - Myocardial infarction | 152 (11.2%) | 62 (10.4%) | 0.64 | 20 (4.6%) | 12 (9.7%) | 0.06 | 65 (4.3%) | 3 (1.6%) | 0.13 |
| - Heart failure | 70 (5.2%) | 23 (3.9%) | 0.25 | 20 (4.6%) | 19 (8.1%) | 0.21 | 53 (3.5%) | 4 (2.2%) | 0.49 |
| - GERD | 381 (28.2%) | 171 (28.7%) | 0.84 | 65 (15.0%) | 22 (17.7%) | 0.56 | 187 (12.3%) | 22 (12.0%) | 1.00 |
| - Stroke or TIA | 87 (6.4%) | 36 (6.1%) | 0.83 | 16 (3.7%) | 6 (4.9%) | 0.74 | 9 (2.3%) | 1 (2.2%) | 1.00 |
| mMRC | 1.6±1.4 | 2.2±1.4 | <0.01 | 1.65±1.50 | 2.54±1.41 | 0.41 | 1.6±1.4 | 2.2±1.4 | <0.01 |
| Total SGRQ score | 30.5±21.2 | 46.5±20.8 | <0.01 | 33.5±23.2 | 54.6±22.6 | <0.01 | 29.9±17.7 | 46.2±21.5 | <0.01 |
| - Symptom | 34.8±23.1 | 61.9±19.9 | <0.01 | 37.2±24.5 | 65.8±21.8 | <0.01 | 39.3±18.6 | 61.6±18.3 | <0.01 |
| - Activity | 44.7±29.5 | 58.3±26.0 | <0.01 | 47.5±30.0 | 66.7±26.1 | <0.01 | 41.6±22.8 | 54.8±26.1 | <0.01 |
| - Impact | 21.1±19.1 | 35.3±21.4 | <0.01 | 24.4±22.6 | 44.4±24.8 | <0.01 | 20.4±18.2 | 36.4±23.7 | <0.01 |
| CAT score | 12.7±8.0 | 18.3±8.5 | <0.01 | 14.3±8.7 | 21.6±6.8 | <0.01 | 13.7±7.6 | 20.2±8.7 | <0.01 |
| - CAT1 (cough) | 1.9±1.2 | 3.0±1.4 | <0.01 | 2.0±1.4 | 3.0±1.1 | <0.01 | 1.6±1.3 | 3.0±1.4 | <0.01 |
| - CAT2 (sputum) | 1.7±1.3 | 2.7±1.3 | <0.01 | 1.6±1.5 | 2.5±1.4 | <0.01 | 1.9±1.4 | 3.1±1.4 | <0.01 |
| 6MWT (m) | 403.9±125.3 | 381.6±120.9 | <0.01 | 358.1±127.7 | 343.8±114.8 | 0.27 | 383.7±117.1 | 384.7±98.9 | 0.91 |
| Depression | 66 (9.0%) | 45 (16.5%) | <0.01 | 21 (10.4%) | 9 (17.3%) | 0.26 | 232 (25.9%) | 40 (41.2%) | <0.01 |
| Anxiety | 63 (8.6%) | 41 (15.1%) | <0.01 | 24 (11.9%) | 8 (15.4%) | 0.67 | 144 (19.0%) | 19 (22.9%) | 0.48 |
| Blood eosinophil count | 213.8±152.9 | 222.7±221.5 | 0.55 | 173.9±150.8 | 182.0±157.1 | 0.74 | 232.9±263.3 | 235.8±242.2 | 0.90 |
| GOLD stage |  |  | <0.01 |  |  | 0.07 |  |  | <0.01 |
| - I (FEV1 ≥80%) | 289 (21.4%) | 68 (11.4%) |  | 84 (19.4%) | 12 (9.7%) |  | 181 (11.9%) | 11 (6.0%) |  |
| - II (FEV1 50-80%) | 532 (39.3%) | 243 (40.8%) |  | 194 (44.9%) | 61 (49.2%) |  | 862 (56.5%) | 76 (41.5%) |  |
| - III (FEV1 30-50%) | 342 (25.3%) | 188 (31.5%) |  | 99 (22.9%) | 36 (29.0%) |  | 402 (26.4%) | 82 (44.8%) |  |
| - IV (FEV1 <30%) | 189 (14.0%) | 97 (16.3%) |  | 55 (12.7%) | 15 (12.1%) |  | 80 (5.2%) | 14 (7.7%) |  |
| postBD FEV1 (L) | 2.0±0.9 | 1.8±0.8 | <0.01 | 1.9±0.8 | 1.7±0.7 | 0.12 | 1.7±0.6 | 1.5±0.5 | <0.01 |
| postBD FEV1 (%) | 58.5±24.0 | 52.8±21.3 | <0.01 | 59.6±22.8 | 54.9±20.1 | 0.04 | 59.0±18.0 | 50.6±16.8 | <0.01 |
| postBD FVC (L) | 3.7±1.0 | 3.6±1.0 | 0.11 | 3.3±0.9 | 3.3±0.9 | 0.84 | 3.4±0.8 | 3.4±0.8 | 0.49 |
| postBD FVC (%) | 82.0±19.8 | 79.6±20.4 | 0.01 | 82.5±21.0 | 80.9±18.9 | 0.44 | 81.4±16.4 | 80.6±16.4 | 0.53 |
| postBD FEV1/FVC | 0.52±0.14 | 0.49±0.13 | <0.01 | 0.55±0.12 | 0.52±0.12 | 0.02 | 0.51±0.12 | 0.45±0.12 | <0.01 |
| DLco | 69.6±24.0 | 66.5±22.8 | 0.09 | 57.5±20.3 | 53.9±20.2 | 0.31 | 63.6±21.0 | 58.0±18.1 | <0.01 |
| Emphysema on CT | 824 (64.3%) | 367 (65.2%) | 0.76 | 203 (51.7%) | 65 (59.1%) | 0.20 | 379 (48.3%) | 55 (55.6%) | 0.21 |
| Medications |  |  | 0.16 |  |  | 0.26 |  |  | 0.04 |
| - no inhaler | 170 (34.8%) | 66 (30.1%) |  | 53 (39.3%) | 14 (31.8%) |  | 364 (23.9%) | 39 (21.3%) |  |
| - LABA or LAMA | 59 (12.1%) | 28 (12.8%) |  | 18 (13.3%) | 6 (13.6%) |  | 380 (24.9%) | 38 (20.8%) |  |
| - LABA/LAMA | 7 (1.4%) | 2 (0.9%) |  | 0 (0%) | 0 (0%) |  | 278 (18.2%) | 32 (17.5%) |  |
| - ICS/LABA | 60 (12.3%) | 42 (19.2%) |  | 26 (19.3%) | 5 (11.4%) |  | 182 (11.9%) | 17 (9.3%) |  |
| - Triple therapy | 192 (39.3%) | 81 (37.0%) |  | 38 (28.1%) | 19 (43.2%) |  | 322 (21.1%) | 57 (31.1%) |  |
| Past exacerbation | 373 (27.6%) | 239 (40.1%) | <0.01 | 104 (24.1%) | 52 (41.9%) | <0.01 | 288 (19.5%) | 50 (28.1%) | 0.01 |
| Past severe exacerbation | 205 (15.2%) | 126 (21.1%) | <0.01 | 93 (21.5%) | 49 (39.5%) | <0.01 | 136 (9.2%) | 26 (14.6%) | 0.03 |

Data are presented as n (%) or mean ± SD

Demographic data in COPDGene was based on phase I database, except for CAT score and HADS score, which were based on phase II database.

All demographic data in KOCOSS was based on the data at the baseline of the study.

BMI Body mass index, DM Diabetes mellitus, HTN Hypertension, GERD Gastroesophageal reflux disease, CT Computed tomography, BDI Beck Depression Inventory, BAI Beck Anxiety Inventory, mMRC modified Medical Research Council, , CAT COPD Assessment Test, 6MWT 6-minute walk distance test, ACO Asthma-COPD overlap

LAMA long-acting muscarinic antagonist, LABA long-acting beta2-agonist, ICS inhaled corticosteroids

Table S6. Difference of clinical characteristics between CB and non-CB by three different race (CAT definition) in male patients

|  | NHW | | | AA | | | Asian | | |
| --- | --- | --- | --- | --- | --- | --- | --- | --- | --- |
|  | Non-CB  (n=742, 72.3%) | CB  (n=284, 27.7%) | P-value | Non-CB  (n=182, 71.1%) | CB  (n=74, 28.9%) | P-value | Non-CB  (n=1339, 77.0%) | CB  (n=399, 23.0%) | P-value |
| Age | 64.8±7.6 | 63.4±8.4 | 0.01 | 58.0±7.2 | 57.1±7.4 | 0.39 | 69.3±7.6 | 68.1±7.7 | <0.01 |
| Smoking status |  |  | <0.01 |  |  | 0.59 |  |  | <0.01 |
| -Ex-smoker | 515 (69.4%) | 153 (53.9%) |  | 52 (28.6%) | 18 (24.3%) |  | 994 (74.2%) | 248 (62.2%) |  |
| -Current smoker | 227 (30.6%) | 131 (46.1%) |  | 130 (71.4%) | 56 (75.7%) |  | 345 (25.8%) | 151 (37.8%) |  |
| Smoking pack-year | 53.3±27.4 | 59.3±28.4 | <0.01 | 44.8±22.1 | 46.9±26.4 | 0.53 | 44.4±24.0 | 47.1±25.5 | 0.053 |
| BMI | 28.6±5.1 | 28.4±5.2 | 0.51 | 27.0±5.7 | 26.3±5.4 | 0.37 | 23.0±3.4 | 22.5±3.4 | <0.01 |
| Comorbidities |  |  |  |  |  |  |  |  |  |
| - DM | 101 (13.6%) | 35 (12.3%) | 0.66 | 22 (12.1%) | 9 (12.2%) | 0.11 | 240 (17.9%) | 62 (15.5%) | 0.30 |
| - HTN | 352 (47.5%) | 118 (41.5%) | 0.10 | 84 (46.2%) | 36 (48.6%) | 0.82 | 553 (41.3%) | 138 (34.6%) | 0.02 |
| - Myocardial infarction | 76 (10.2%) | 22 (7.7%) | 0.27 | 10 (5.5%) | 9 (12.2%) | 0.11 | 58 (4.3%) | 13 (3.3%) | 0.42 |
| - Heart failure | 22 (3.0%) | 14 (4.9%) | 0.18 | 3 (1.6%) | 6 (8.1%) | 0.03 | 45 (3.4%) | 14 (3.5%) | 1.00 |
| - GERD | 221 (29.8%) | 93 (32.7%) | 0.41 | 25 (13.7%) | 16 (21.6%) | 0.17 | 161 (12.0%) | 52 (13.0%) | 0.65 |
| - Stroke or TIA | 45 (6.1%) | 17 (6.0%) | 1.00 | 4 (2.2%) | 4 (5.4%) | 0.35 | 9 (2.6%) | 1 (1.2%) | 0.73 |
| mMRC | 1.2±1.3 | 1.9±1.4 | <0.01 | 1.6±1.5 | 1.9±1.4 | 0.19 | 1.2±0.8 | 1.6±0.9 | <0.01 |
| Total SGRQ score | 25.1±19.3 | 39.8±20.7 | <0.01 | 33.5±24.7 | 43.3±23.5 | <0.01 | 27.3±15.8 | 46.0±21.0 | <0.01 |
| - Symptom | 32.4±22.9 | 51.8±24.2 | <0.01 | 38.5±27.1 | 52.9±25.1 | <0.01 | 36.6±17.4 | 58.5±18.0 | <0.01 |
| - Activity | 36.5±27.4 | 51.9±26.3 | <0.01 | 45.6±31.2 | 56.1±28.8 | <0.01 | 39.6±21.8 | 54.2±25.7 | <0.01 |
| - Impact | 16.5±16.8 | 29.4±20.0 | <0.01 | 25.0±23.6 | 33.1±23.5 | <0.01 | 17.5±15.6 | 37.4±23.2 | <0.01 |
| CAT score | 11.2±6.9 | 22.2±7.0 | <0.01 | 12.8±7.9 | 23.1±6.4 | <0.01 | 12.0±6.5 | 22.7±7.2 | <0.01 |
| - CAT1 (cough) | 1.6±1.0 | 3.8±0.8 | <0.01 | 1.6±1.2 | 3.6±0.8 | <0.01 | 1.2±1.0 | 3.6±0.8 | <0.01 |
| - CAT2 (sputum) | 1.3±1.0 | 3.6±0.7 | <0.01 | 1.1±1.0 | 3.6±0.8 | <0.01 | 1.5±1.1 | 3.7±0.8 | <0.01 |
| 6MWT (m) | 443.3±106.9 | 406.1±112.4 | <0.01 | 385.6±115.6 | 336.8±118.4 | <0.01 | 387.0±115.9 | 374.8±111.3 | 0.10 |
| Depression | 56 (7.7%) | 55 (19.9%) | <0.01 | 19 (10.6%) | 11 (15.1%) | 0.43 | 195 (24.3%) | 81 (39.1%) | <0.01 |
| Anxiety | 55 (7.6%) | 49 (17.8%) | <0.01 | 18 (10.0%) | 14 (19.2%) | 0.08 | 112 (16.1%) | 53 (33.1%) | <0.01 |
| Blood eosinophil count | 213.1±157.3 | 224.7±212.6 | 0.42 | 174.5±150.6 | 178.5±155.7 | 0.85 | 233.3±269.9 | 229.4±222.2 | 0.79 |
| GOLD stage |  |  | <0.01 |  |  | 0.01 |  |  | <0.01 |
| - I (FEV1 ≥80%) | 203 (27.4%) | 31 (10.9%) |  | 41 (22.5%) | 5 (6.8%) |  | 170 (12.7%) | 28 (7.0%) |  |
| - II (FEV1 50-80%) | 331 (44.6%) | 128 (45.1%) |  | 90 (49.5%) | 39 (52.7%) |  | 747 (55.8%) | 200 (50.1%) |  |
| - III (FEV1 30-50%) | 162 (21.8%) | 103 (36.3%) |  | 41 (22.5%) | 22 (29.7%) |  | 364 (27.2%) | 134 (33.6%) |  |
| - IV (FEV1 <30%) | 46 (6.2%) | 22 (7.7%) |  | 10 (5.5%) | 8 (10.8%) |  | 57 (4.3%) | 37 (9.3%) |  |
| postBD FEV1 (L) | 2.2±0.8 | 1.9±0.7 | <0.01 | 2.0±0.7 | 1.8±0.6 | 0.02 | 1.8±0.6 | 1.6±0.6 | <0.01 |
| postBD FEV1 (%) | 64.6±22.0 | 55.7±19.3 | <0.01 | 64.1±20.8 | 56.5±18.9 | <0.01 | 59.5±18.0 | 53.3±17.5 | <0.01 |
| postBD FVC (L) | 3.9±1.0 | 3.6±1.0 | <0.01 | 3.5±0.9 | 3.2±0.8 | 0.01 | 3.4±0.8 | 3.3±0.8 | 0.04 |
| postBD FVC (%) | 86.2±18.2 | 81.3±18.5 | <0.01 | 87.1±18.8 | 79.4±17.1 | <0.01 | 81.8±16.3 | 79.9±16.5 | 0.04 |
| postBD FEV1/FVC | 0.55±0.12 | 0.51±0.12 | <0.01 | 0.57±0.11 | 0.55±0.11 | 0.21 | 0.51±0.11 | 0.48±0.12 | <0.01 |
| DLco | 70.5±23.9 | 63.6±22.5 | <0.01 | 58.9±20.7 | 50.8±18.1 | 0.01 | 63.8±20.7 | 60.4±20.6 | <0.01 |
| Emphysema on CT | 430 (60.0) | 187 (69.0%) | 0.01 | 94 (53.7%) | 36 (54.5%) | 1.00 | 320 (47.6%) | 118 (53.4%) | 0.16 |
| Medications |  |  | 0.04 |  |  | 0.74 |  |  | <0.01 |
| - no inhaler | 179 (37.4%) | 57 (26.9%) |  | 47 (37.9%) | 20 (37.7%) |  | 300 (22.4%) | 110 (27.6%) |  |
| - LABA or LAMA | 62 (12.9%) | 25 (11.8%) |  | 15 (12.1%) | 9 (17.0%) |  | 341 (25.5%) | 86 (21.6%) |  |
| - LABA/LAMA | 6 (1.3%) | 3 (1.4%) |  | 0 (0%) | 0 (0%) |  | 261 (19.5%) | 56 (14.0%) |  |
| - ICS/LABA | 61 (12.7%) | 41 (19.3%) |  | 21 (16.9%) | 10 (18.9%) |  | 158 (11.8%) | 43 (10.8%) |  |
| - Triple therapy | 171 (35.7%) | 86 (40.6%) |  | 41 (33.1%) | 14 (26.4%) |  | 279 (20.8%) | 104 (26.1%) |  |
| Past exacerbation | 171 (23.0%) | 96 (33.8%) | <0.01 | 38 (20.9%) | 29 (39.2%) | <0.01 | 243 (18.8%) | 98 (25.2%) | <0.01 |
| Past severe exacerbation | 74 (10.0%) | 57 (20.1%) | <0.01 | 41 (22.5%) | 22 (29.7%) | 0.29 | 112 (8.7%) | 51 (13.1%) | 0.01 |

Data are presented as n (%) or mean ± SD

Demographic data in COPDGene was based on phase I database, except for CAT score and HADS score, which were based on phase II database.

All demographic data in KOCOSS was based on the data at the baseline of the study.

BMI Body mass index, DM Diabetes mellitus, HTN Hypertension, GERD Gastroesophageal reflux disease, CT Computed tomography, BDI Beck Depression Inventory, BAI Beck Anxiety Inventory, mMRC modified Medical Research Council, , CAT COPD Assessment Test, 6MWT 6-minute walk distance test, ACO Asthma-COPD overlap

LAMA long-acting muscarinic antagonist, LABA long-acting beta2-agonist, ICS inhaled corticosteroids

Table S7. Baseline characteristics of COPDGene and KOCOSS data in age- and sex- matched population

|  | NHW vs AA | | | AA vs Asian | | | NHW vs Asian | | |
| --- | --- | --- | --- | --- | --- | --- | --- | --- | --- |
|  | NHW  (N=1013) | AA  (N=1013) | P-value | AA  (n=449) | Asian  (n=449) | P-value | NHW  (N=1511) | Asian  (N=1511) | P-vslue |
| Age | 58.7±8.2 | 58.7±8.2 | 0.95 | 62.2±7.7 | 62.2±8.3 | 0.99 | 67.4±7.0 | 67.5±7.1 | 0.68 |
| Sex (male) | 541 (53.4%) | 553 (54.6%) | 0.62 | 392 (87.3%) | 398 (88.6%) | 0.61 | 1463 (96.8%) | 1463 (96.8%) | 1.00 |
| Smoking status |  |  | <0.01 |  |  | <0.01 |  |  | 0.55 |
| -Ex-smoker | 466 (46.0%) | 352 (34.7%) |  | 170 (37.9%) | 269 (59.9%) |  | 1064 (70.4%) | 1048 (69.4%) |  |
| -Current smoker | 547 (54.0%) | 661 (65.3%) |  | 279 (62.1%) | 180 (40.1%) |  | 447 (29.6%) | 463 (30.6%) |  |
| Smoking pack-year | 49.6±24.4 | 42.2±23.6 | <0.01 | 45.7±24.2 | 40.8±21.6 | <0.01 | 60.5±31.0 | 44.2±23.3 | <0.01 |
| BMI | 27.9±6.2 | 27.9±6.6 | 0.92 | 26.8±5.8 | 23.1±3.6 | <0.01 | 28.3±5.5 | 22.9±3.4 | <0.01 |
| Comorbidities |  |  |  |  |  |  |  |  |  |
| - DM | 95 (9.4%) | 155 (15.3%) | <0.01 | 69 (15.4%) | 71 (15.8%) | 0.93 | 236 (15.6%) | 258 (17.1%) | 0.30 |
| - HTN | 411 (40.6%) | 542 (53.5%) | <0.01 | 253 (56.3%) | 148 (33.0%) | <0.01 | 788 (52.2%) | 581 (38.5%) | <0.01 |
| - Myocardial infarction | 69 (6.8%) | 59 (5.8%) | 0.41 | 24 (5.3%) | 18 (4.0%) | <0.01 | 177 (11.7%) | 60 (4.0%) | <0.01 |
| - Heart failure | 42 (4.1%) | 50 (4.9%) | 0.46 | 23 (5.1%) | 12 (2.7%) | 0.09 | 75 (5.0%) | 50 (3.3%) | 0.03 |
| - GERD | 304 (30.0%) | 198 (19.5%) | <0.01 | 85 (18.9%) | 60 (13.4%) | 0.03 | 437 (28.9%) | 189 (12.5%) | <0.01 |
| - Stroke or TIA | 39 (3.8%) | 57 (5.6%) | 0.08 | 21 (4.7%) | 2 (1.9%) | 0.29 | 108 (7.2%) | 11 (3.1%) | <0.01 |
| mMRC | 1.9±1.5 | 2.1±1.5 | <0.01 | 2.9±1.5 | 1.2±0.9 | <0.01 | 1.8±1.4 | 1.3±0.9 | <0.01 |
| Total SGRQ score | 37.4±23.2 | 40.1±24.2 | 0.01 | 39.1±23.4 | 29.3±18.3 | <0.01 | 35.1±21.8 | 31.3±18.7 | <0.01 |
| - Symptom | 45.4±26.6 | 44.0±26.3 | 0.25 | 43.7±25.4 | 42.1±20.3 | 0.30 | 41.8±25.0 | 41.8±19.7 | 0.96 |
| - Activity | 50.7±29.0 | 55.5±29.9 | <0.01 | 54.2±28.9 | 38.6±23.2 | <0.01 | 49.7±28.7 | 42.1±23.4 | <0.01 |
| - Impact | 27.5±22.2 | 30.0±24.0 | 0.01 | 29.2±23.4 | 20.0±18.9 | <0.01 | 24.9±20.3 | 21.9±19.4 | <0.01 |
| CAT score | 15.0±8.8 | 16.5±8.8 | <0.01 | 15.1±8.6 | 14.1±7.9 | 0.12 | 14.0±8.2 | 14.4±8.1 | 0.20 |
| - CAT1 (cough) | 2.4±1.5 | 2.3±1.4 | 0.52 | 2.1±1.4 | 1.8±1.4 | <0.01 | 2.1±1.3 | 1.8±1.4 | <0.01 |
| - CAT2 (sputum) | 1.9±1.4 | 1.9±1.5 | 0.72 | 1.8±1.5 | 2.1±1.4 | 0.03 | 1.9±1.4 | 2.1±1.4 | <0.01 |
| 6MWT (m) | 402.2±120.3 | 336.4±125.5 | <0.01 | 338.3±126.8 | 401.7±110.3 | <0.01 | 385.1±124. | 2389.7±111.8 | 0.33 |
| CB (classic definition) | 319 (31.5%) | 212 (20.9%) | <0.01 | 97 (21.6%) | 61 (13.9%) | <0.01 | 423 (28.0%) | 168 (11.3%) | <0.01 |
| CB (CAT definition) | 155 (29.2%) | 157 (30.8%) | 0.62 | 57 (27.4%) | 107 (23.8%) | 0.38 | 209 (26.4%) | 353 (23.4%) | 0.12 |
| Depression | 70 (13.5%) | 55 (10.9%) | 0.23 | 15 (7.3%) | 80 (28.3%) | <0.01 | 74 (9.6%) | 242 (27.3%) | <0.01 |
| Anxiety | 95 (18.3%) | 80 (15.8%) | 0.32 | 20 (9.8%) | 54 (23.1%) | <0.01 | 64 (8.3%) | 150 (20.3%) | <0.01 |
| Blood eosinophil count | 203.4±135.9 | 166.3±134.7 | <0.01 | 180.2±157.5 | 244.7±295.1 | <0.01 | 216.7±178. | 5235.1±267.2 | 0.07 |
| GOLD stage |  |  | 0.15 |  |  | <0.01 |  |  | <0.01 |
| - I (FEV1 ≥80%) | 198 (19.5%) | 176 (17.4%) |  | 71 (15.8%) | 53 (11.8%) |  | 253 (16.7%) | 176 (11.7%) |  |
| - II (FEV1 50-80%) | 433 (42.7%) | 479 (47.3%) |  | 192 (42.8%) | 265 (59.0%) |  | 601 (39.8%) | 827 (54.8%) |  |
| - III (FEV1 30-50%) | 248 (24.5%) | 244 (24.1%) |  | 126 (28.1%) | 104 (23.2%) |  | 424 (28.1%) | 421 (27.9%) |  |
| - IV (FEV1 <30%) | 134 (13.2%) | 114 (11.3%) |  | 60 (13.4%) | 27 (6.0%) |  | 233 (15.4%) | 86 (5.7%) |  |
| postBD FEV1 (L) | 1.9±0.9 | 1.6±0.7 | <0.01 | 1.6±0.7 | 1.9±0.6 | <0.01 | 1.8±0.8 | 1.7±0.6 | 0.09 |
| postBD FEV1 (%) | 58.9±23.2 | 59.0±22.2 | 0.92 | 56.8±22.5 | 59.4±18.1 | 0.051 | 55.6±23.1 | 58.2±18.1 | <0.01 |
| postBD FVC (L) | 3.4±1.1 | 2.8±1.0 | <0.01 | 3.0±0.9 | 3.5±0.9 | <0.01 | 3.5±1.0 | 3.4±0.8 | 0.22 |
| postBD FVC (%) | 82.6±20.3 | 82.8±21.2 | 0.84 | 80.6±20.5 | 85.0±16.4 | <0.01 | 80.5±19.9 | 82.4±16.3 | <0.01 |
| postBD FEV1/FVC | 0.53±0.13 | 0.55±0.12 | 0.01 | 0.53±0.13 | 0.52±0.11 | 0.14 | 0.50±0.14 | 0.51±0.12 | 0.21 |
| DLco | 70.6±23.4 | 57.2±20.3 | <0.01 | 54.3±19.6 | 65.5±20.9 | <0.01 | 66.2±22.9 | 63.7±20.8 | 0.02 |
| Emphysema on CT | 492 (52.7%) | 447 (49.2%) | 0.14 | 236 (57.8%) | 101 (40.7%) | <0.01 | 969 (68.0%) | 359 (46.7%) | <0.01 |
| Medications |  |  | 0.80 |  |  | <0.01 |  |  | <0.01 |
| - no inhaler | 137 (36.6%) | 131 (34.2%) |  | 52 (34.9%) | 119 (26.5%) |  | 164 (30.0%) | 359 (23.8%) |  |
| - LABA or LAMA | 45 (12.0%) | 50 (13.1%) |  | 21 (14.1%) | 123 (27.4%) |  | 67 (12.2%) | 383 (25.3%) |  |
| - LABA/LAMA | 3 (0.8%) | 1 (0.3%) |  | 0 (0.0%) | 75 (16.7%) |  | 7 (1.3%) | 261 (17.3%) |  |
| - ICS/LABA | 67 (17.9%) | 71 (18.5%) |  | 26 (17.4%) | 53 (11.8%) |  | 82 (15.0%) | 186 (12.3%) |  |
| - ICS/LABA/LAMA | 122 (32.6%) | 130 (33.9%) |  | 50 (33.6%) | 79 (17.6%) |  | 227 (41.5%) | 322 (21.3%) |  |
| Past exacerbation | 353 (34.8%) | 318 (31.4%) | 0.11 | 136 (30.3%) | 73 (17.0%) | <0.01 | 489 (32.4%) | 281 (19.3%) | <0.01 |
| Past severe exacerbation | 205 (20.2%) | 281 (27.7%) | <0.01 | 107 (23.8%) | 38 (8.8%) | <0.01 | 254 (16.8%) | 136 (9.3%) | <0.01 |

Data are presented as n (%) or mean ± SD

Demographic data in COPDGene was based on phase I database, except for CAT score and HADS score, which were based on phase II database.

All demographic data in KOCOSS was based on the data at the baseline of the study.

BMI Body mass index, DM Diabetes mellitus, HTN Hypertension, GERD Gastroesophageal reflux disease, CT Computed tomography, BDI Beck Depression Inventory, BAI Beck Anxiety Inventory, mMRC modified Medical Research Council, , CAT COPD Assessment Test, 6MWT 6-minute walk distance test, ACO Asthma-COPD overlap

LAMA long-acting muscarinic antagonist, LABA long-acting beta2-agonist, ICS inhaled corticosteroids

Table S8. Difference of clinical characteristics between CB and non-CB by three different race (classic definition) in age- and sex- matched population

1) NHW vs AA

|  | NWH | | | AA | | |
| --- | --- | --- | --- | --- | --- | --- |
|  | Non-CB  (n=694, 68.5%) | CB  (n=319, 31.5%) | P-value | Non-CB  (n=801,79.1%)) | CB  (n=212, 20.9%)) | P-value |
| Age | 59.6±8.4 | 56.8±7.2 | <0.01 | 58.9±8.3 | 57.9±7.7 | 0.14 |
| Sex (male) | 345 (49.7%) | 196 (61.4%) | <0.01 | 429 (53.6%) | 124 (58.5%) | 0.23 |
| Smoking status |  |  | <0.01 |  |  | <0.01 |
| -Ex-smoker | 375 (54.0%) | 91 (28.5%) |  | 298 (37.2%) | 54 (25.5%) |  |
| -Current smoker | 319 (46.0%) | 228 (71.5%) |  | 503 (62.8%) | 158 (74.5%) |  |
| Smoking pack-year | 48.5±24.4 | 52.1±24.4 | 0.03 | 41.6±23.2 | 44.5±25.1 | 0.11 |
| BMI | 27.9±6.2 | 27.8±6.3 | 0.79 | 27.9±6.5 | 27.9±7.3 | 0.94 |
| Comorbidities |  |  |  |  |  |  |
| - DM | 63 (9.1%) | 32 (10.0%) | 0.71 | 125 (15.6%) | 30 (14.2%) | 0.68 |
| - HTN | 281 (40.5%) | 130 (40.8%) | 0.99 | 422 (52.7%) | 120 (56.6%) | 0.35 |
| - Myocardial infarction | 40 (5.8%) | 29 (9.1%) | 0.07 | 44 (5.5%) | 15 (7.1%) | 0.48 |
| - Heart failure | 26 (3.7%) | 16 (5.0%) | 0.44 | 37 (4.6%) | 13 (6.1%) | 0.47 |
| - GERD | 203 (29.3%) | 101 (31.7%) | 0.48 | 150 (18.7%) | 48 (22.6%) | 0.24 |
| - Stroke or TIA | 23 (3.3%) | 16 (5.0%) | 0.26 | 46 (5.7%) | 11 (5.2%) | 0.90 |
| mMRC | 1.6±1.4 | 2.3±1.4 | <0.01 | 1.9±1.5 | 2.6±1.3 | <0.01 |
| Total SGRQ score | 31.7±21.8 | 49.8±21.1 | <0.01 | 36.4±23.6 | 54.0±21.1 | <0.01 |
| - Symptom | 36.5±24.6 | 64.7±19.6 | <0.01 | 38.7±25.0 | 63.9±21.1 | <0.01 |
| - Activity | 46.0±29.0 | 61.2±26.0 | <0.01 | 52.3±30.2 | 67.9±25.5 | <0.01 |
| - Impact | 22.2±20.1 | 39.0±22.1 | <0.01 | 26.6±23.1 | 43.1±22.9 | <0.01 |
| CAT score | 12.9±8.1 | 20.3±8.4 | <0.01 | 15.3±8.8 | 21.6±7.3 | <0.01 |
| - CAT1 (cough) | 2.0±1.3 | 3.3±1.4 | <0.01 | 2.1±1.4 | 3.0±1.2 | <0.01 |
| - CAT2 (sputum) | 1.6±1.3 | 2.8±1.4 | <0.01 | 1.7±1.5 | 2.7±1.4 | <0.01 |
| 6MWT (m) | 408.4±122.5 | 388.5±114.4 | 0.02 | 339.6±128.0 | 324.2±114.7 | 0.12 |
| Depression | 34 (9.1%) | 36 (24.7%) | <0.01 | 42 (10.3%) | 13 (13.3%) | 0.50 |
| Anxiety | 57 (15.3%) | 38 (26.0%) | <0.01 | 64 (15.7%) | 16 (16.3%) | 0.99 |
| Blood eosinophil count | 208.5±142.2 | 190.0±117.2 | 0.14 | 165.2±131.4 | 170.8±148.2 | 0.71 |
| GOLD stage |  |  | <0.01 |  |  | 0.04 |
| - I (FEV1 ≥80%) | 156 (22.5%) | 42 (13.2%) |  | 150 (18.7%) | 26 (12.3%) |  |
| - II (FEV1 50-80%) | 295 (42.5%) | 138 (43.3%) |  | 367 (45.8%) | 112 (52.8%) |  |
| - III (FEV1 30-50%) | 161 (23.2%) | 87 (27.3%) |  | 188 (23.5%) | 56 (26.4%) |  |
| - IV (FEV1 <30%) | 82 (11.8%) | 52 (16.3%) |  | 96 (12.0%) | 18 (8.5%) |  |
| postBD FEV1 (L) | 1.9±0.9 | 1.8±0.8 | 0.11 | 1.6±0.7 | 1.6±0.6 | 0.73 |
| postBD FEV1 (%) | 60.9±23.7 | 54.7±21.5 | <0.01 | 59.4±22.6 | 57.5±20.5 | 0.27 |
| postBD FVC (L) | 3.4±1.1 | 3.4±1.1 | 0.60 | 2.8±1.0 | 2.9±0.9 | 0.33 |
| postBD FVC (%) | 83.7±20.2 | 80.3±20.3 | 0.01 | 82.8±21.6 | 82.6±19.7 | 0.89 |
| postBD FEV1/FVC | 0.54±0.13 | 0.51±0.13 | <0.01 | 0.5±0.12 | 0.54±0.12 | 0.21 |
| DLco | 71.7±23.2 | 67.8±23.8 | 0.12 | 57.5±20.6 | 56.0±19.0 | 0.58 |
| Emphysema on CT | 346 (53.9%) | 146 (50.2%) | 0.33 | 350 (48.5%) | 97 (51.9%) | 0.46 |
| Medications |  |  | 0.18 |  |  | 0.44 |
| - no inhaler | 101 (39.8%) | 36 (30.0%) |  | 105 (35.2%) | 26 (30.6%) |  |
| - LABA or LAMA | 28 (11.0%) | 17 (14.2%) |  | 40 (13.4%) | 10 (11.8%) |  |
| - LABA/LAMA | 3 (1.2%) | 0 (0.0%) |  | 1 (0.3%) | 0 (0.0%) |  |
| - ICS/LABA | 40 (15.7%) | 27 (22.5%) |  | 58 (19.5%) | 13 (15.3%) |  |
| - Triple therapy | 82 (32.3%) | 40 (33.3%) |  | 94 (31.5%) | 36 (42.4%) |  |
| Past exacerbation | 211 (30.4%) | 142 (44.5%) | <0.01 | 225 (28.1%) | 93 (43.9%) | <0.01 |
| Past severe exacerbation | 123 (17.7%) | 82 (25.7%) | <0.01 | 197 (24.6%) | 84 (39.6%) | <0.01 |

Data are presented as n (%) or mean ± SD

Demographic data in COPDGene was based on phase I database, except for CAT score and HADS score, which were based on phase II database.

All demographic data in KOCOSS was based on the data at the baseline of the study.

BMI Body mass index, DM Diabetes mellitus, HTN Hypertension, GERD Gastroesophageal reflux disease, CT Computed tomography, BDI Beck Depression Inventory, BAI Beck Anxiety Inventory, mMRC modified Medical Research Council, , CAT COPD Assessment Test, 6MWT 6-minute walk distance test, ACO Asthma-COPD overlap

LAMA long-acting muscarinic antagonist, LABA long-acting beta2-agonist, ICS inhaled corticosteroids

2) AA vs Asian

|  | AA | | | Asian | | |
| --- | --- | --- | --- | --- | --- | --- |
|  | Non-CB  (n=352, 78.3%) | CB  (n=97, 21.7%) | P-value | Non-CB  (n=377,86.1%) | CB  (n=61, 13.9%) | P-value |
| Age | 62.6±7.8 | 61.1±7.2 | 0.10 | 62.0±8.2 | 63.9±8.3 | 0.10 |
| Sex | 306 (86.9%) | 86 (88.7%) | 0.78 | 331 (87.8%) | 56 (91.8%) | 0.49 |
| Smoking status |  |  | 0.052 |  |  | 0.16 |
| -Ex-smoker | 142 (40.3%) | 28 (28.9%) |  | 231 (61.3%) | 31 (50.8%) |  |
| -Current smoker | 210 (59.7%) | 69 (71.1%) |  | 146 (38.7%) | 30 (49.2%) |  |
| Smoking pack-year | 44.3±22.5 | 50.8±29.3 | 0.045 | 40.1±20.9 | 43.8±23.9 | 0.21 |
| BMI | 26.9±5.6 | 26.7±6.3 | 0.82 | 23.1±3.6 | 22.5±3.3 | 0.21 |
| Comorbidities |  |  |  |  |  |  |
| - DM | 55 (15.6%) | 14 (14.4%) | 0.90 | 60 (15.9%) | 10 (16.4%) | 1.00 |
| - HTN | 195 (55.4%) | 58 (59.8%) | 0.51 | 123 (32.6%) | 24 (39.3%) | 0.38 |
| - Myocardial infarction | 16 (4.5%) | 8 (8.2%) | 0.24 | 16 (4.2%) | 1 (1.6%) | 0.54 |
| - Heart failure | 16 (4.5%) | 7 (7.2%) | 0.43 | 10 (2.7%) | 2 (3.3%) | 1.00 |
| - GERD | 63 (17.9%) | 22 (22.7%) | 0.36 | 52 (13.8%) | 7 (11.5%) | 0.77 |
| - Stroke or TIA | 16 (4.5%) | 5 (5.2%) | 1.00 | 1 (1.1%) | 1 (5.0%) | 0.82 |
| mMRC | 1.9±1.5 | 2.5±1.3 | <0.01 | 1.2±0.9 | 1.5±0.8 | <0.01 |
| Total SGRQ score | 35.7±22.5 | 51.8±22.4 | <0.01 | 27.9±17.5 | 38.7±20.4 | <0.01 |
| - Symptom | 38.5±23.8 | 62.4±22.1 | <0.01 | 39.3±18.7 | 59.3±21.2 | <0.01 |
| - Activity | 51.3±29.0 | 64.7±26.1 | <0.01 | 37.6±22.8 | 46.8±24.5 | <0.01 |
| - Impact | 25.8±22.1 | 41.3±24.2 | <0.01 | 18.9±17.9 | 27.3±23.0 | <0.01 |
| CAT score | 13.8±8.6 | 20.6±6.5 | <0.01 | 13.4±7.7 | 18.6±8.0 | <0.01 |
| - CAT1 (cough) | 1.9±1.4 | 2.9±1.2 | <0.01 | 1.6±1.3 | 2.8±1.4 | <0.01 |
| - CAT2 (sputum) | 1.6±1.4 | 2.6±1.3 | <0.01 | 1.9±1.3 | 3.0±1.4 | <0.01 |
| 6MWT (m) | 339.7±131.5 | 333.2±108.7 | 0.62 | 399.8±112.8 | 412.6±99.5 | 0.46 |
| Depression | 10 (6.1%) | 5 (11.9%) | 0.34 | 63 (27.0%) | 16 (38.1%) | 0.20 |
| Anxiety | 14 (8.6%) | 6 (14.3%) | 0.41 | 47 (24.2%) | 6 (17.6%) | 0.54 |
| Blood eosinophil count | 183.5±157.8 | 167.6±157.9 | 0.56 | 259.3±315.9 | 174.5±124.8 | <0.01 |
| GOLD stage |  |  | 0.24 |  |  | 0.04 |
| - I (FEV1 ≥80%) | 62 (17.6%) | 9 (9.3%) |  | 48 (12.7%) | 3 (4.9%) |  |
| - II (FEV1 50-80%) | 148 (42.0%) | 44 (45.4%) |  | 229 (60.7%) | 32 (52.5%) |  |
| - III (FEV1 30-50%) | 95 (27.0%) | 31 (32.0%) |  | 78 (20.7%) | 21 (34.4%) |  |
| - IV (FEV1 <30%) | 47 (13.4%) | 13 (13.4%) |  | 22 (5.8%) | 5 (8.2%) |  |
| postBD FEV1 (L) | 1.7±0.7 | 1.6±0.7 | 0.29 | 1.9±0.6 | 1.6±0.6 | <0.01 |
| postBD FEV1 (%) | 57.5±22.7 | 54.3±21.9 | 0.21 | 60.4±18.0 | 53.7±17.0 | <0.01 |
| postBD FVC (L) | 3.0±0.9 | 2.9±0.9 | 0.65 | 3.6±0.9 | 3.4±0.8 | 0.25 |
| postBD FVC (%) | 81.0±20.6 | 78.9±20.1 | 0.37 | 85.1±16.5 | 83.4±16.2 | 0.45 |
| postBD FEV1/FVC | 0.5±0.1 | 0.5±0.1 | 0.29 | 0.53±0.11 | 0.48±0.12 | <0.01 |
| DLco | 55.0±20.4 | 51.5±15.7 | 0.37 | 65.7±21.1 | 63.8±20.9 | 0.54 |
| Emphysema on CT | 187 (57.7%) | 49 (58.3%) | 1.00 | 80 (37.7%) | 19 (57.6%) | 0.049 |
| Medications |  |  | 0.39 |  |  | 0.49 |
| - no inhaler | 41 (36.9%) | 11 (28.9%) |  | 106 (28.1%) | 11 (18.0%) |  |
| - LABA or LAMA | 16 (14.4%) | 5 (13.2%) |  | 104 (27.6%) | 18 (29.5%) |  |
| - LABA/LAMA | 0 (0%) | 0 (0%) |  | 61 (16.2%) | 10 (16.4%) |  |
| - ICS/LABA | 21 (18.9%) | 5 (13.2%) |  | 44 (11.7%) | 8 (13.1%) |  |
| - Triple therapy | 33 (29.7%) | 17 (44.7%) |  | 62 (16.4%) | 14 (23.0%) |  |
| Past exacerbation | 94 (26.7%) | 42 (43.3%) | <0.01 | 59 (16.3%) | 13 (22.4%) | 0.34 |
| Past severe exacerbation | 71 (20.2%) | 36 (37.1%) | <0.01 | 31 (8.6%) | 7 (12.1%) | 0.54 |

Data are presented as n (%) or mean ± SD

Demographic data in COPDGene was based on phase I database, except for CAT score and HADS score, which were based on phase II database.

All demographic data in KOCOSS was based on the data at the baseline of the study.

BMI Body mass index, DM Diabetes mellitus, HTN Hypertension, GERD Gastroesophageal reflux disease, CT Computed tomography, BDI Beck Depression Inventory, BAI Beck Anxiety Inventory, mMRC modified Medical Research Council, , CAT COPD Assessment Test, 6MWT 6-minute walk distance test, ACO Asthma-COPD overlap

LAMA long-acting muscarinic antagonist, LABA long-acting beta2-agonist, ICS inhaled corticosteroids

3) NHW vs Asian

|  | NHW | | | Asian | | | |
| --- | --- | --- | --- | --- | --- | --- | --- |
|  | Non-CB  (n=1088, 72.0%) | CB  (n=423, 28.0%) | P-value | Non-CB  (n=1315, 88.7%) | CB  (n=168,11.4%) | | P-value |
| Age | 67.7±7.0 | 66.5±7.1 | <0.01 | 67.6±7.1 | 67.1±6.9 | 0.44 | |
| Sex | 1049 (96.4%) | 414 (97.9%) | 0.20 | 1271 (96.7%) | 164 (97.6%) | 0.66 | |
| Smoking status |  |  | <0.01 |  |  | 0.37 | |
| -Ex-smoker | 823 (75.6%) | 241 (57.0%) |  | 918 (69.8%) | 111 (66.1%) |  | |
| -Current smoker | 265 (24.4%) | 182 (43.0%) |  | 397 (30.2%) | 57 (33.9%) |  | |
| Smoking pack-year | 59.0±30.7 | 64.3±31.7 | <0.01 | 43.6±22.8 | 48.4±26.4 | 0.03 | |
| BMI | 28.4±5.4 | 28.2±5.6 | 0.59 | 23.0±3.4 | 22.1±3.1 | <0.01 | |
| Comorbidities |  |  |  |  |  |  | |
| - DM | 177 (16.3%) | 59 (13.9%) | 0.30 | 226 (17.2%) | 29 (17.3%) | 1.00 | |
| - HTN | 574 (52.8%) | 214 (50.6%) | 0.47 | 517 (39.3%) | 59 (35.1%) | 0.33 | |
| - Myocardial infarction | 133 (12.2%) | 44 (10.4%) | 0.37 | 54 (4.1%) | 3 (1.8%) | 0.21 | |
| - Heart failure | 60 (5.5%) | 15 (3.5%) | 0.15 | 44 (3.3%) | 4 (2.4%) | 0.66 | |
| - GERD | 323 (29.7%) | 114 (27.0%) | 0.34 | 166 (12.6%) | 19 (11.3%) | 0.72 | |
| - Stroke or TIA | 80 (7.4%) | 28 (6.6%) | 0.71 | 10 (3.2%) | 1 (2.4%) | 1.00 | |
| mMRC | 1.6±1.4 | 2.2±1.4 | <0.01 | 1.2±0.9 | 1.7±1.0 | <0.01 | |
| Total SGRQ score | 31.1±20.8 | 45.6±21.1 | <0.01 | 29.6±17.6 | 45.0±21.5 | <0.01 | |
| - Symptom | 34.5±22.9 | 60.5±19.8 | <0.01 | 39.3±18.4 | 61.5±18.6 | <0.01 | |
| - Activity | 46.3±29.0 | 58.5±26.1 | <0.01 | 40.8±22.7 | 52.6±25.9 | <0.01 | |
| - Impact | 21.4±18.7 | 33.8±21.8 | <0.01 | 20.2±18.1 | 35.5±23.6 | <0.01 | |
| CAT score | 12.8±7.8 | 17.5±8.4 | <0.01 | 13.7±7.7 | 20.3±8.7 | <0.01 | |
| - CAT1 (cough) | 1.8±1.2 | 2.8±1.3 | <0.01 | 1.6±1.3 | 3.1±1.4 | <0.01 | |
| - CAT2 (sputum) | 1.6±1.3 | 2.6±1.3 | <0.01 | 1.9±1.3 | 3.2±1.4 | <0.01 | |
| 6MWT (m) | 392.8±124.5 | 364.9±121.1 | <0.01 | 389.9±113.3 | 386.4±102.3 | 0.72 | |
| Depression | 50 (8.7%) | 24 (12.6%) | 0.15 | 198 (25.5%) | 40 (43.0%) | <0.01 | |
| Anxiety | 46 (8.0%) | 18 (9.4%) | 0.63 | 131 (20.2%) | 17 (21.5%) | 0.90 | |
| Blood eosinophil count | 210.7±153.5 | 234.7±237.9 | 0.19 | 236.6±275.3 | 229.6±215.3 | 0.72 | |
| GOLD stage |  |  | <0.01 |  |  | <0.01 | |
| - I (FEV1 ≥80%) | 210 (19.3%) | 43 (10.2%) |  | 159 (12.1%) | 11 (6.5%) |  | |
| - II (FEV1 50-80%) | 433 (39.8%) | 168 (39.7%) |  | 747 (56.8%) | 72 (42.9%) |  | |
| - III (FEV1 30-50%) | 288 (26.5%) | 136 (32.2%) |  | 335 (25.5%) | 72 (42.9%) |  | |
| - IV (FEV1 <30%) | 157 (14.4%) | 76 (18.0%) |  | 73 (5.6%) | 13 (7.7%) |  | |
| postBD FEV1 (L) | 1.8±0.8 | 1.7±0.8 | <0.01 | 1.8±0.6 | 1.5±0.5 | <0.01 | |
| postBD FEV1 (%) | 57.3±23.7 | 51.3±20.9 | <0.01 | 59.1±18.0 | 51.0±16.5 | <0.01 | |
| postBD FVC (L) | 3.5±1.0 | 3.4±1.0 | 0.15 | 3.4±0.8 | 3.4±0.8 | 0.24 | |
| postBD FVC (%) | 81.1±19.7 | 78.9±20.2 | 0.049 | 82.4±16.3 | 81.0±16.2 | 0.30 | |
| postBD FEV1/FVC | 0.51±0.14 | 0.48±0.13 | <0.01 | 0.5±0.1 | 0.5±0.1 | <0.01 | |
| DLco | 67.0±23.4 | 63.8±21.3 | 0.13 | 64.4±21.1 | 58.7±18.2 | <0.01 | |
| Emphysema on CT | 682 (66.5%) | 287 (71.9%) | 0.06 | 306 (45.7%) | 49 (53.8%) | 0.18 | |
| Medications |  |  | 0.03 |  |  | 0.02 | |
| - no inhaler | 128 (32.3%) | 36 (23.8%) |  | 315 (24.0%) | 36 (21.4%) |  | |
| - LABA or LAMA | 47 (11.9%) | 20 (13.2%) |  | 341 (25.9%) | 33 (19.6%) |  | |
| - LABA/LAMA | 5 (1.3%) | 2 (1.3%) |  | 228 (17.3%) | 28 (16.7%) |  | |
| - ICS/LABA | 48 (12.1%) | 34 (22.5%) |  | 166 (12.6%) | 18 (10.7%) |  | |
| - Triple therapy | 168 (42.4%) | 59 (39.1%) |  | 265 (20.2%) | 53 (31.5%) |  | |
| Past exacerbation | 317 (29.1%) | 172 (40.7%) | <0.01 | 236 (18.6%) | 42 (25.8%) | 0.04 | |
| Past severe exacerbation | 167 (15.3%) | 87 (20.6%) | 0.02 | 112 (8.8%) | 23 (14.1%) | 0.04 | |

Data are presented as n (%) or mean ± SD

Demographic data in COPDGene was based on phase I database, except for CAT score and HADS score, which were based on phase II database.

All demographic data in KOCOSS was based on the data at the baseline of the study.

BMI Body mass index, DM Diabetes mellitus, HTN Hypertension, GERD Gastroesophageal reflux disease, CT Computed tomography, BDI Beck Depression Inventory, BAI Beck Anxiety Inventory, mMRC modified Medical Research Council, , CAT COPD Assessment Test, 6MWT 6-minute walk distance test, ACO Asthma-COPD overlap

LAMA long-acting muscarinic antagonist, LABA long-acting beta2-agonist, ICS inhaled corticosteroids

Table S9. Difference of clinical characteristics between CB and non-CB by three different race (CAT definition) in age- and sex- matched population

1) NHW vs AA

|  | NWH | | | AA | | |
| --- | --- | --- | --- | --- | --- | --- |
|  | Non-CB  (n=375, 70.8%) | CB  (n=155, 29.2%) | P-value | Non-CB  (n=352, 69.2%) | CB  (n=157, 30.8%) | P-value |
| Age | 59.8±8.1 | 57.1±7.8 | <0.01 | 58.7±7.8 | 57.1±7.2 | 0.03 |
| Sex | 192 (51.2%) | 84 (54.2%) | 0.60 | 181 (51.4%) | 73 (46.5%) | 0.35 |
| Smoking status |  |  | <0.01 |  |  | 0.53 |
| -Ex-smoker | 208 (55.5%) | 53 (34.2%) |  | 110 (31.2%) | 44 (28.0%) |  |
| -Current smoker | 167 (44.5%) | 102 (65.8%) |  | 242 (68.8%) | 113 (72.0%) |  |
| Smoking pack-year | 46.4±21.4 | 51.7±25.4 | 0.02 | 41.9±21.9 | 41.6±23.4 | 0.89 |
| BMI | 28.7±6.1 | 27.8±5.9 | 0.13 | 28.1±6.6 | 28.2±6.7 | 0.82 |
| Comorbidities |  |  |  |  |  |  |
| - DM | 35 (9.3%) | 13 (8.4%) | 0.86 | 51 (14.5%) | 24 (15.3%) | 0.92 |
| - HTN | 154 (41.1%) | 59 (38.1%) | 0.59 | 179 (50.9%) | 86 (54.8%) | 0.47 |
| - Myocardial infarction | 26 (6.9%) | 8 (5.2%) | 0.57 | 18 (5.1%) | 12 (7.6%) | 0.36 |
| - Heart failure | 9 (2.4%) | 12 (7.7%) | <0.01 | 6 (1.7%) | 10 (6.4%) | 0.01 |
| - GERD | 122 (32.5%) | 50 (32.3%) | 1.00 | 64 (18.2%) | 36 (22.9%) | 0.26 |
| - Stroke or TIA | 13 (3.5%) | 10 (6.5%) | 0.19 | 12 (3.4%) | 10 (6.4%) | 0.20 |
| mMRC | 1.4±1.4 | 2.0±1.4 | <0.01 | 1.7±1.5 | 2.3±1.4 | <0.01 |
| Total SGRQ score | 27.6±20.6 | 43.9±22.2 | <0.01 | 33.4±23.1 | 45.5±22.9 | <0.01 |
| - Symptom | 34.8±24.7 | 55.4±25.4 | <0.01 | 37.7±25.9 | 52.6±25.0 | <0.01 |
| - Activity | 40.0±27.7 | 56.1±26.3 | <0.01 | 47.8±30.1 | 60.0±27.1 | <0.01 |
| - Impact | 18.5±18.5 | 33.6±22.3 | <0.01 | 23.9±21.7 | 35.1±23.7 | <0.01 |
| CAT score | 11.8±7.2 | 22.9±7.3 | <0.01 | 13.3±7.9 | 23.7±6.4 | <0.01 |
| - CAT1 (cough) | 1.7±1.1 | 3.9±0.9 | <0.01 | 1.7±1.2 | 3.6±0.8 | <0.01 |
| - CAT2 (sputum) | 1.2±1.0 | 3.6±0.7 | <0.01 | 1.1±1.1 | 3.6±0.8 | <0.01 |
| 6MWT (m) | 433.2±107.1 | 401.7±115.3 | <0.01 | 364.6±110.4 | 341.4±129.4 | 0.053 |
| Depression | 32 (8.8%) | 38 (24.8%) | <0.01 | 35 (10.0%) | 20 (12.8%) | 0.43 |
| Anxiety | 59 (16.2%) | 36 (23.5%) | 0.06 | 40 (11.4%) | 40 (25.6%) | <0.01 |
| Blood eosinophil count | 200.6±135.6 | 210.1±136.9 | 0.48 | 167.0±132.1 | 165.2±141.1 | 0.89 |
| GOLD stage |  |  | <0.01 |  |  | 0.01 |
| - I (FEV1 ≥80%) | 97 (25.9%) | 17 (11.0%) |  | 77 (21.9%) | 17 (10.8%) |  |
| - II (FEV1 50-80%) | 176 (46.9%) | 84 (54.2%) |  | 186 (52.8%) | 84 (53.5%) |  |
| - III (FEV1 30-50%) | 82 (21.9%) | 46 (29.7%) |  | 76 (21.6%) | 47 (29.9%) |  |
| - IV (FEV1 <30%) | 20 (5.3%) | 8 (5.2%) |  | 13 (3.7%) | 9 (5.7%) |  |
| postBD FEV1 (L) | 2.0±0.8 | 1.8±0.7 | <0.01 | 1.7±0.7 | 1.5±0.6 | <0.01 |
| postBD FEV1 (%) | 65.1±21.6 | 58.4±18.4 | <0.01 | 64.6±19.5 | 59.0±18.7 | <0.01 |
| postBD FVC (L) | 3.5±1.1 | 3.4±1.0 | 0.22 | 3.0±0.9 | 2.7±0.8 | <0.01 |
| postBD FVC (%) | 86.7±18.9 | 82.6±18.6 | 0.02 | 87.8±19.0 | 81.0±17.9 | <0.01 |
| postBD FEV1/FVC | 0.57±0.11 | 0.54±0.11 | 0.03 | 0.57±0.10 | 0.57±0.10 | 0.49 |
| DLco | 72.4±23.0 | 66.2±23.9 | 0.01 | 58.4±20.5 | 54.2±19.6 | 0.07 |
| Emphysema on CT | 171 (48.9%) | 78 (53.1%) | 0.45 | 152 (46.6%) | 64 (44.8%) | 0.78 |
| Medications |  |  | 0.54 |  |  | 0.76 |
| - no inhaler | 98 (40.8%) | 39 (31.5%) |  | 92 (36.4%) | 39 (32.0%) | 92 (36.4%) |
| - LABA or LAMA | 28 (11.7%) | 17 (13.7%) |  | 30 (11.9%) | 19 (15.6%) | 30 (11.9%) |
| - LABA/LAMA | 2 (0.8%) | 1 (0.8%) |  | 1 (0.4%) | 0 (0.0%) | 1 (0.4%) |
| - ICS/LABA | 42 (17.5%) | 25 (20.2%) |  | 48 (19.0%) | 23 (18.9%) | 48 (19.0%) |
| - Triple therapy | 70 (29.2%) | 42 (33.9%) |  | 82 (32.4%) | 41 (33.6%) | 82 (32.4%) |
| Past exacerbation | 98 (26.1%) | 58 (37.4%) | 0.01 | 85 (24.1%) | 57 (36.3%) | <0.01 |
| Past severe exacerbation | 54 (14.4%) | 33 (21.3%) | 0.07 | 80 (22.7%) | 47 (29.9%) | 0.10 |

Data are presented as n (%) or mean ± SD

Demographic data in COPDGene was based on phase I database, except for CAT score and HADS score, which were based on phase II database.

All demographic data in KOCOSS was based on the data at the baseline of the study.

BMI Body mass index, DM Diabetes mellitus, HTN Hypertension, GERD Gastroesophageal reflux disease, CT Computed tomography, BDI Beck Depression Inventory, BAI Beck Anxiety Inventory, mMRC modified Medical Research Council, , CAT COPD Assessment Test, 6MWT 6-minute walk distance test, ACO Asthma-COPD overlap

LAMA long-acting muscarinic antagonist, LABA long-acting beta2-agonist, ICS inhaled corticosteroids

2) AA vs Asian

|  | AA | | | Asian | | |
| --- | --- | --- | --- | --- | --- | --- |
|  | Non-CB  (n=151, 72.9%) | CB  (n=57, 27.1%) | P-value | Non-CB  (n=342,76.2%) | CB  (n=107, 23.8%) | P-value |
| Age | 61.2±7.3 | 61.1±7.4 | 0.92 | 62.7±8.5 | 60.8±7.5 | 0.04 |
| Sex | 131 (86.8%) | 47 (82.5%) | 0.571 | 303 (88.6%) | 95 (88.8%) | 1.00 |
| Smoking status |  |  | 1.00 |  |  | <0.01 |
| -Ex-smoker | 52 (34.4%) | 19 (33.3%) |  | 225 (65.8%) | 44 (41.1%) |  |
| -Current smoker | 99 (65.6%) | 38 (66.7%) |  | 117 (34.2%) | 63 (58.9%) |  |
| Smoking pack-year | 43.6±21.1 | 45.6±23.8 | 0.558 | 40.2±20.7 | 42.5±24.2 | 0.39 |
| BMI | 26.7±5.2 | 27.2±5.6 | 0.62 | 23.1±3.7 | 22.8±3.3 | 0.46 |
| Comorbidities |  |  |  |  |  |  |
| - DM | 19 (12.6%) | 6 (10.5%) | 0.87 | 58 (17.0%) | 13 (12.1%) | 0.30 |
| - HTN | 75 (49.7%) | 35 (61.4%) | 0.18 | 115 (33.6%) | 33 (30.8%) | 0.68 |
| - Myocardial infarction | 5 (3.3%) | 8 (14.0%) | 0.01 | 17 (5.0%) | 1 (0.9%) | 0.12 |
| - Heart failure | 2 (1.3%) | 6 (10.5%) | <0.01 | 9 (2.6%) | 3 (2.8%) | 1.00 |
| - GERD | 26 (17.2%) | 15 (26.3%) | 0.20 | 44 (12.9%) | 16 (15.0%) | 0.70 |
| - Stroke or TIA | 3 (2.0%) | 4 (7.0%) | 0.18 | 1 (1.2%) | 1 (3.8%) | 0.98 |
| mMRC | 1.6±1.5 | 2.1±1.4 | 0.049 | 1.2±0.9 | 1.4±0.9 | 0.01 |
| Total SGRQ score | 32.3±22.3 | 43.9±22.8 | <0.01 | 25.8±15.8 | 39.9±21.0 | <0.01 |
| - Symptom | 36.7±24.7 | 53.8±25.1 | <0.01 | 37.5±18.2 | 56.4±19.9 | <0.01 |
| - Activity | 45.7±28.9 | 57.4±28.9 | 0.01 | 36.0±21.6 | 46.7±25.9 | <0.01 |
| - Impact | 23.3±21.3 | 33.2±22.5 | <0.01 | 16.3±15.6 | 31.3±23.1 | <0.01 |
| CAT score | 12.3±7.6 | 22.7±6.1 | <0.01 | 11.7±6.4 | 21.7±7.3 | <0.01 |
| - CAT1 (cough) | 1.6±1.2 | 3.5±0.8 | <0.01 | 1.2±1.0 | 3.6±0.8 | <0.01 |
| - CAT2 (sputum) | 1.1±1.0 | 3.6±0.8 | <0.01 | 1.6±1.2 | 3.6±0.8 | <0.01 |
| 6MWT (m) | 375.4±115.4 | 335.4±118.5 | 0.03 | 396.2±112.7 | 418.6±101.3 | 0.12 |
| Depression | 10 (6.7%) | 5 (8.9%) | 0.81 | 55 (24.4%) | 25 (43.1%) | <0.01 |
| Anxiety | 12 (8.1%) | 8 (14.3%) | 0.28 | 37 (20.0%) | 17 (34.7%) | 0.048 |
| Blood eosinophil count | 179.1±157.0 | 183.1±160.3 | 0.87 | 256.1±325.3 | 207.8±158.1 | 0.06 |
| GOLD stage |  |  | 0.04 |  |  | 0.08 |
| - I (FEV1 ≥80%) | 32 (21.2%) | 4 (7.0%) |  | 45 (13.2%) | 8 (7.5%) |  |
| - II (FEV1 50-80%) | 73 (48.3%) | 30 (52.6%) |  | 204 (59.6%) | 61 (57.0%) |  |
| - III (FEV1 30-50%) | 39 (25.8%) | 16 (28.1%) |  | 77 (22.5%) | 27 (25.2%) |  |
| - IV (FEV1 <30%) | 7 (4.6%) | 7 (12.3%) |  | 16 (4.7%) | 11 (10.3%) |  |
| postBD FEV1 (L) | 1.8±0.7 | 1.7±0.6 | 0.08 | 1.9±0.6 | 1.8±0.6 | 0.07 |
| postBD FEV1 (%) | 63.2±20.7 | 56.9±20.0 | 0.048 | 60.5±17.9 | 56.0±18.1 | 0.03 |
| postBD FVC (L) | 3.2±0.9 | 3.0±0.8 | 0.04 | 3.6±0.9 | 3.5±0.8 | 0.55 |
| postBD FVC (%) | 86.6±18.4 | 79.4±18.8 | 0.01 | 85.1±16.4 | 84.7±16.5 | 0.83 |
| postBD FEV1/FVC | 0.56±0.11 | 0.55±0.11 | 0.43 | 0.53±0.11 | 0.50±0.12 | 0.02 |
| DLco | 56.1±20.4 | 49.7±16.7 | 0.06 | 66.6±21.2 | 62.3±19.6 | 0.08 |
| Emphysema on CT | 82 (56.6%) | 28 (53.8%) | 0.86 | 73 (38.6%) | 28 (47.5%) | 0.29 |
| Medications |  |  | 0.36 |  |  | 0.49 |
| - no inhaler | 41 (40.2%) | 11 (25.0%) |  | 85 (24.9%) | 34 (31.8%) |  |
| - LABA or LAMA | 13 (12.7%) | 8 (18.2%) |  | 100 (29.2%) | 23 (21.5%) |  |
| - LABA/LAMA | 0 (0%) | 0 (0%) |  | 57 (16.7%) | 18 (16.8%) |  |
| - ICS/LABA | 17 (16.7%) | 9 (20.5%) |  | 41 (12.0%) | 12 (11.2%) |  |
| - Triple therapy | 31 (30.4%) | 16 (36.4%) |  | 59 (17.3%) | 20 (18.7%) |  |
| Past exacerbation | 30 (19.9%) | 26 (45.6%) | <0.01 | 50 (15.3%) | 23 (22.3%) | 0.13 |
| Past severe exacerbation | 30 (19.9%) | 18 (31.6%) | 0.053 | 26 (8.0%) | 12 (11.7%) | 0.34 |

Data are presented as n (%) or mean ± SD

Demographic data in COPDGene was based on phase I database, except for CAT score and HADS score, which were based on phase II database.

All demographic data in KOCOSS was based on the data at the baseline of the study.

BMI Body mass index, DM Diabetes mellitus, HTN Hypertension, GERD Gastroesophageal reflux disease, CT Computed tomography, BDI Beck Depression Inventory, BAI Beck Anxiety Inventory, mMRC modified Medical Research Council, , CAT COPD Assessment Test, 6MWT 6-minute walk distance test, ACO Asthma-COPD overlap

LAMA long-acting muscarinic antagonist, LABA long-acting beta2-agonist, ICS inhaled corticosteroids

3) NHW vs Asian

|  | NHW | | | Asian | | |
| --- | --- | --- | --- | --- | --- | --- |
|  | Non-CB  (n=583, 73.6%) | CB  (n=209, 26.4%) | P-value | Non-CB  (n=1157, 76.6%) | CB  (n=353, 23.4%) | P-value |
| Age | 67.4±6.5 | 66.5±7.1 | 0.073 | 67.8±7.1 | 66.7±7.0 | 0.01 |
| Sex | 560 (96.1%) | 205 (98.1%) | 0.244 | 1121 (96.9%) | 341 (96.6%) | 0.92 |
| Smoking status |  |  | <0.01 |  |  | <0.01 |
| -Ex-smoker | 443 (76.0%) | 131 (62.7%) |  | 835 (72.2%) | 213 (60.3%) |  |
| -Current smoker | 140 (24.0%) | 78 (37.3%) |  | 322 (27.8%) | 140 (39.7%) |  |
| Smoking pack-year | 54.4±28.5 | 60.9±30.1 | <0.01 | 43.6±22.6 | 46.3±25.3 | 0.07 |
| BMI | 28.6±5.0 | 28.9±5.1 | 0.37 | 23.0±3.4 | 22.5±3.3 | 0.02 |
| Comorbidities |  |  |  |  |  |  |
| - DM | 86 (14.8%) | 32 (15.3%) | 0.94 | 200 (17.3%) | 58 (16.4%) | 0.77 |
| - HTN | 295 (50.7%) | 93 (44.5%) | 0.15 | 458 (39.6%) | 123 (34.8%) | 0.12 |
| - Myocardial infarction | 65 (11.1%) | 16 (7.7%) | 0.20 | 48 (4.1%) | 12 (3.4%) | 0.64 |
| - Heart failure | 16 (2.7%) | 9 (4.3%) | 0.38 | 40 (3.5%) | 10 (2.8%) | 0.69 |
| - GERD | 177 (30.4%) | 69 (33.0%) | 0.54 | 144 (12.4%) | 45 (12.7%) | 0.95 |
| - Stroke or TIA | 42 (7.2%) | 14 (6.7%) | 0.93 | 9 (3.2%) | 2 (2.7%) | 1.00 |
| mMRC | 1.2±1.3 | 1.9±1.4 | <0.01 | 1.2±0.8 | 1.6±0.9 | <0.01 |
| Total SGRQ score | 25.0±19.1 | 39.1±20.1 | <0.01 | 26.9±15.4 | 45.6±21.3 | <0.01 |
| - Symptom | 31.7±22.7 | 50.7±23.3 | <0.01 | 36.7±17.1 | 58.5±18.6 | <0.01 |
| - Activity | 37.3±27.4 | 51.7±26.1 | <0.01 | 38.6±21.5 | 53.3±25.9 | <0.01 |
| - Impact | 16.0±16.4 | 28.5±19.4 | <0.01 | 17.2±15.1 | 37.1±23.6 | <0.01 |
| CAT score | 11.1±6.7 | 21.9±6.9 | <0.01 | 11.9±6.4 | 22.8±7.4 | <0.01 |
| - CAT1 (cough) | 1.5±1.0 | 3.7±0.8 | <0.01 | 1.2±1.0 | 3.6±0.8 | <0.01 |
| - CAT2 (sputum) | 1.3±1.1 | 3.5±0.7 | <0.01 | 1.6±1.1 | 3.7±0.8 | <0.01 |
| 6MWT (m) | 438.2±105.7 | 391.9±112.2 | <0.01 | 391.9±113.9 | 382.7±105.0 | 0.23 |
| Depression | 37 (6.5%) | 37 (18.3%) | <0.01 | 165 (23.5%) | 77 (41.6%) | <0.01 |
| Anxiety | 35 (6.2%) | 29 (14.4%) | <0.01 | 99 (16.7%) | 51 (34.5%) | <0.01 |
| Blood eosinophil count | 212.4±158.1 | 228.7±226.0 | 0.35 | 237.5±283.4 | 227.3±208.1 | 0.51 |
| GOLD stage |  |  | <0.01 |  |  | <0.01 |
| - I (FEV1 ≥80%) | 151 (25.9%) | 23 (11.0%) |  | 150 (13.0%) | 26 (7.4%) |  |
| - II (FEV1 50-80%) | 264 (45.3%) | 93 (44.5%) |  | 645 (55.8%) | 181 (51.3%) |  |
| - III (FEV1 30-50%) | 130 (22.3%) | 77 (36.8%) |  | 307 (26.6%) | 114 (32.3%) |  |
| - IV (FEV1 <30%) | 38 (6.5%) | 16 (7.7%) |  | 54 (4.7%) | 32 (9.1%) |  |
| postBD FEV1 (L) | 2.1±0.8 | 1.8±0.7 | <0.01 | 1.8±0.6 | 1.6±0.6 | <0.01 |
| postBD FEV1 (%) | 64.1±22.2 | 55.6±18.9 | <0.01 | 59.5±18.0 | 53.9±17.6 | <0.01 |
| postBD FVC (L) | 3.8±1.0 | 3.5±0.9 | <0.01 | 3.5±0.8 | 3.4±0.8 | 0.06 |
| postBD FVC (%) | 86.0±18.5 | 81.2±17.9 | <0.01 | 82.7±16.2 | 81.1±16.3 | 0.10 |
| postBD FEV1/FVC | 0.54±0.12 | 0.51±0.12 | <0.01 | 0.51±0.12 | 0.48±0.13 | <0.01 |
| DLco | 68.2±23.3 | 60.2±20.7 | <0.01 | 64.7±20.8 | 60.8±20.8 | <0.01 |
| Emphysema on CT | 357 (63.3%) | 144 (72.7%) | 0.02 | 260 (45.1%) | 99 (51.3%) | 0.16 |
| Medications |  |  | 0.11 |  |  | 0.01 |
| - no inhaler | 127 (34.0%) | 37 (23.4%) |  | 263 (22.7%) | 95 (26.9%) |  |
| - LABA or LAMA | 48 (12.8%) | 19 (12.0%) |  | 311 (26.9%) | 72 (20.4%) |  |
| - LABA/LAMA | 5 (1.3%) | 2 (1.3%) |  | 210 (18.2%) | 51 (14.4%) |  |
| - ICS/LABA | 51 (13.6%) | 31 (19.6%) |  | 142 (12.3%) | 44 (12.5%) |  |
| - Triple therapy | 143 (38.2%) | 69 (43.7%) |  | 231 (20.0%) | 91 (25.8%) |  |
| Past exacerbation | 147 (25.2%) | 73 (34.9%) | <0.01 | 199 (17.8%) | 82 (23.9%) | 0.02 |
| Severe exacerbation | 58 (9.9%) | 44 (21.1%) | <0.01 | 93 (8.3%) | 43 (12.5%) | 0.03 |

Data are presented as n (%) or mean ± SD

Demographic data in COPDGene was based on phase I database, except for CAT score and HADS score, which were based on phase II database.

All demographic data in KOCOSS was based on the data at the baseline of the study.

BMI Body mass index, DM Diabetes mellitus, HTN Hypertension, GERD Gastroesophageal reflux disease, CT Computed tomography, BDI Beck Depression Inventory, BAI Beck Anxiety Inventory, mMRC modified Medical Research Council, , CAT COPD Assessment Test, 6MWT 6-minute walk distance test, ACO Asthma-COPD overlap

LAMA long-acting muscarinic antagonist, LABA long-acting beta2-agonist, ICS inhaled corticosteroids

**Figure legends**

Figure S1. Flow chart of patient selection


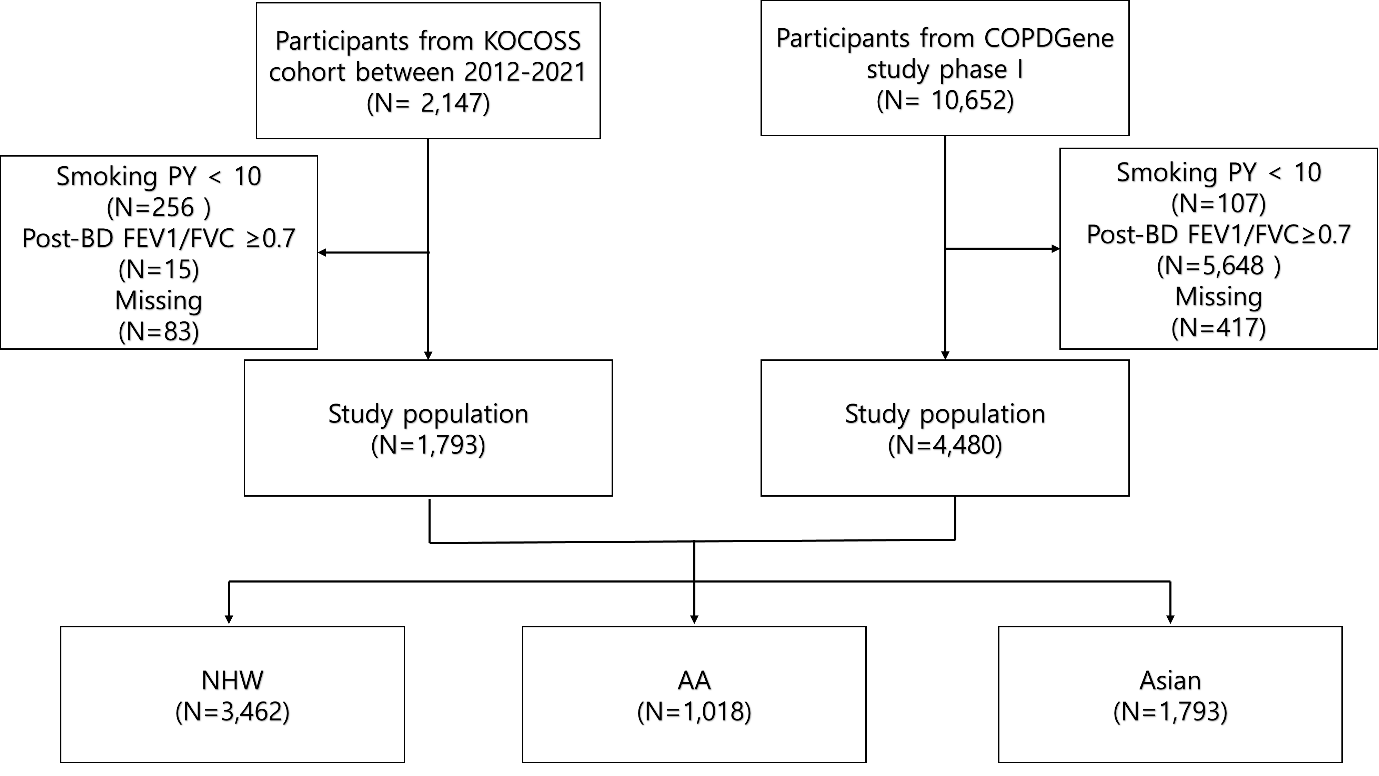


Figure S2. Difference in exacerbation risk between the CB and non-CB groups according to CB definition in male patients


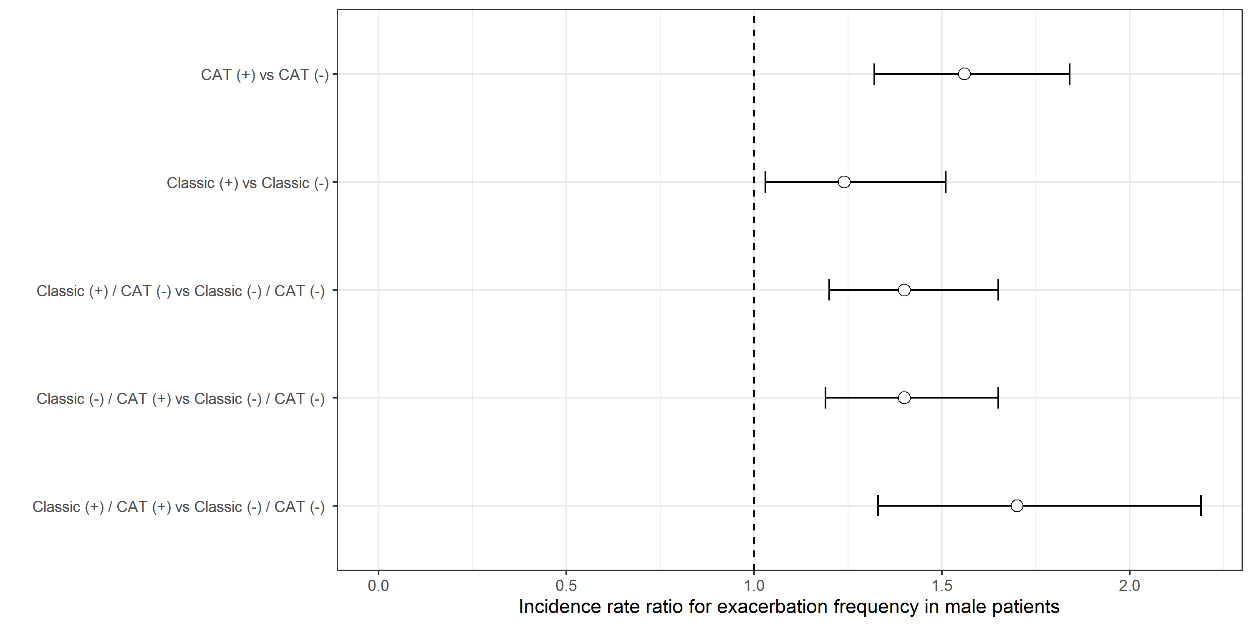


Figure S3. Difference in severe exacerbation risk between the CB and non-CB groups according to CB definition in male patients


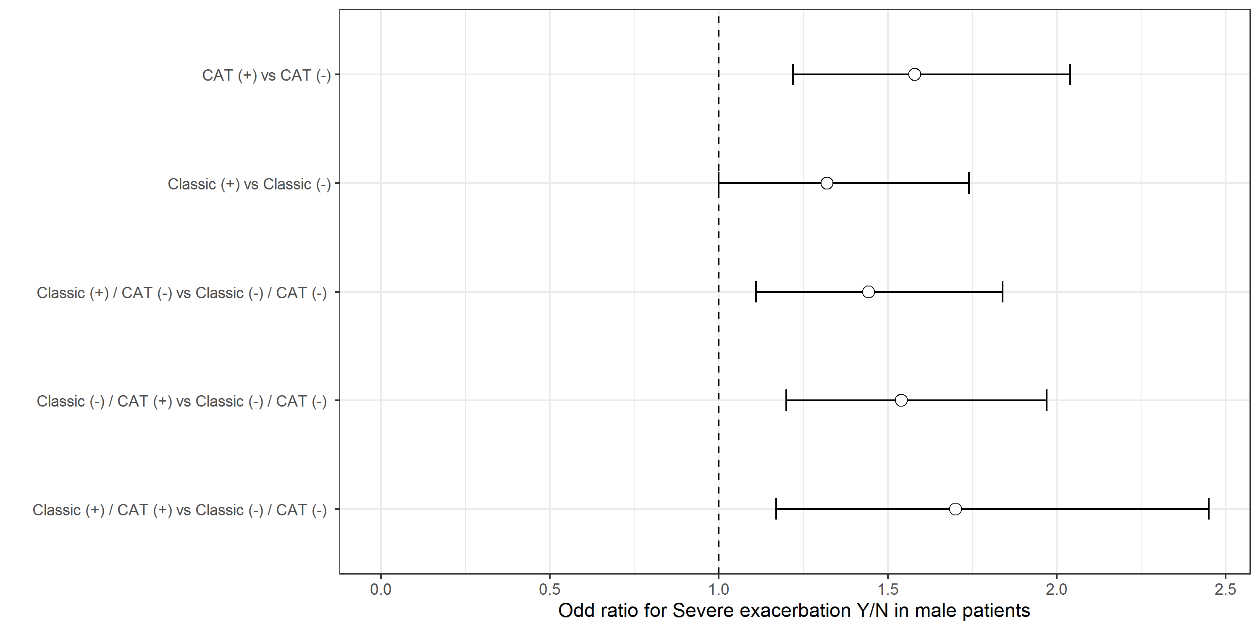


Figure S4. Difference in exacerbation frequency between the CB and non-CB groups according to race and CB definition in male patients


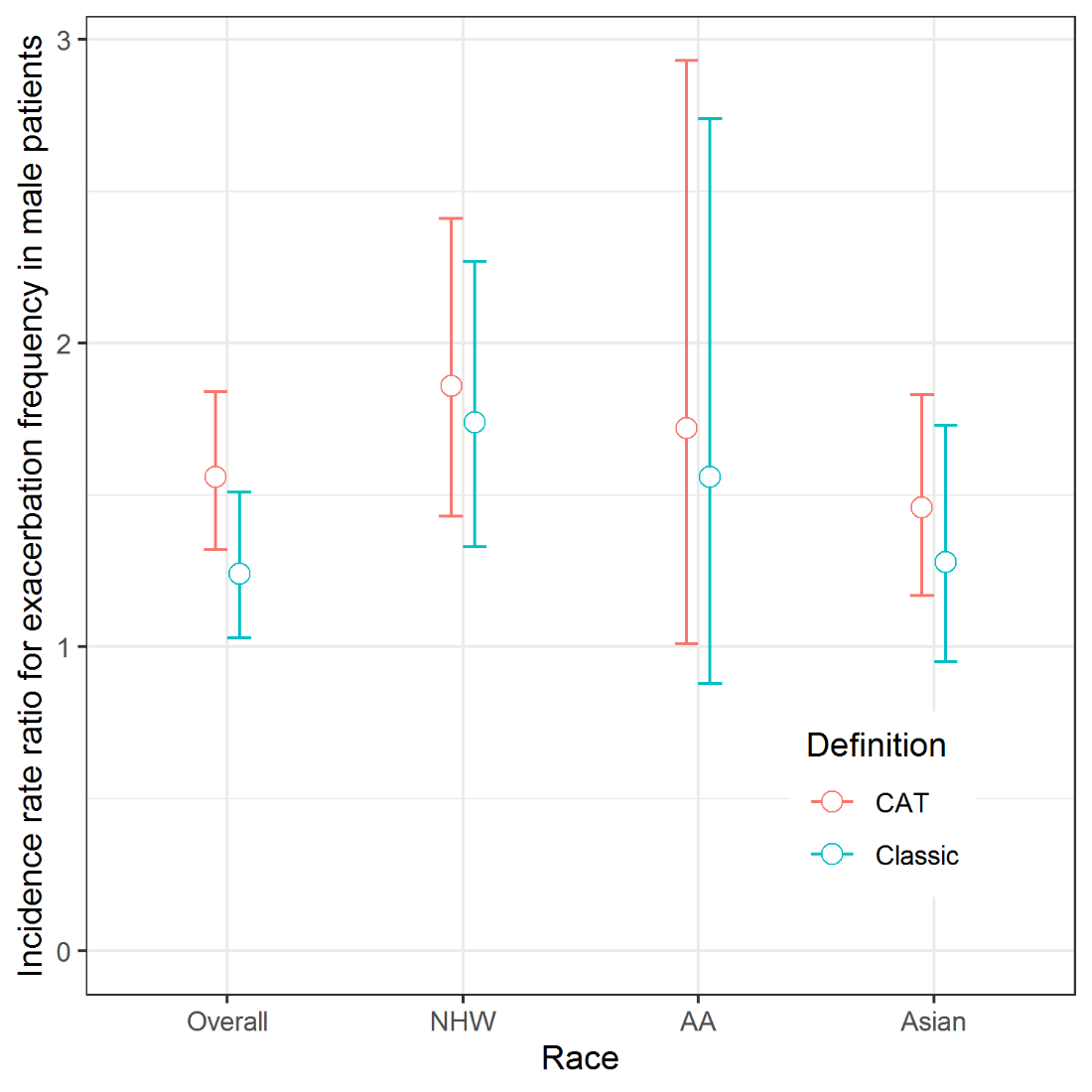

Supplement: Supplementary file 1 — Supplementary Material 1 [file 12890_2024_3100_MOESM1_ESM.docx]
